# The Sub 2-h Official Marathon is Possible: Developing a Drafting Strategy for a Historic Breakthrough in Sports

**DOI:** 10.1186/s40798-024-00802-9

**Published:** 2025-01-27

**Authors:** G. D. Fernandes, Nazir Laureano Gandur, Dioser Santos, Victor Maldonado

**Affiliations:** 1 Mechanical Engineering Department, Texas Tech University, Lubbock, TX USA; 2 Department of Aerospace Engineering, Embry-Riddle Aeronautical University, Prescott, AZ USA

**Keywords:** Drafting, Pacing, Marathon, Eliud Kipchoge, CFD, World record

## Abstract

**Background:**

Drafting for drag reduction is a tactic commonly employed by elite athletes of various sports. The strategy has been adopted by Kenyan runner Eliud Kipchoge on numerous marathon events in the past, including the 2018 and 2022 editions of the Berlin marathon (where Kipchoge set two official world records), as well as in two special attempts to break the 2 h mark for the distance, the Nike Breaking2 (2017) and the INEOS 1:59 Challenge (2019), where Kipchoge used an improved drafting formation to finish in 1:59:40, although that is not recognized as an official record.

**Results:**

In this study, the drag of a realistic model of a male runner is calculated by computational fluid dynamics for a range of velocities. The formations employed in the past by Kipchoge, as well as alternative formations, are analyzed and systematically compared with respect to mechanical power. In a quest to show that running an official marathon in under 2 h is possible, the power analysis is extended to the pacers. We developed a simple drafting and pacing strategy that Kipchoge could have used to run the 2022 Berlin marathon in a surprising 1 h, 59 min and 48 s.

**Conclusions:**

Elite marathon runners can make better use of the pacers to experience reduced drag in races. The associated energy reduction makes it possible to run faster, finishing the race in less time. Using a better drafting strategy and a positive splitting pacing strategy, Kenyan runner Eliud Kipchoge could have broken the sub 2 h barrier in both the 2018 and 2022 editions of Berlin Marathon.

## Background

In most competitive racing sports, drag is a significant physical phenomenon influencing performance [1]. As the athlete moves forward, he or she has to overcome the resistance exerted by the surrounding fluid, such as water or air. It is common practice, both in sports and motorsports, to benefit from the presence of other athletes or vehicles to minimize the aerodynamic resistance. The region of flow behind a bluff body [2] is denominated *wake* and is characterized by lower flow velocity and negative pressure coefficient, which benefit the trailing contestant.

The procedure of employing formations to minimize drag is denominated *drafting* and has been long studied for motorsports [3, 4]. Research regarding the effects of drafting in other sports has been extensive, including in kayaking [5], skating [6], skiing [7] and swimming [8, 9]. Perhaps the effect of drafting was studied the most in cycling. It was shown by computational fluid dynamics (CFD) that large benefits are achievable by drafting, especially in tight formations [10–12]. In CFD studies with two cyclists, drag reductions of up to 27.1% were found for the trailing cyclist [13]. Wind tunnel experiments have also been performed with real cyclists to investigate drafting in a setup of two riders [14].

Studies on the effect of drafting and its relation to metabolic savings on running performance have also been conducted, especially for long distance races, which usually involve lower speeds. The earliest major study on the subject dates back to the 1970s [15] and established a relation between the rate of oxygen uptake $(\dot{V}{O}_{2})$ necessary for a runner to overcome air resistance and sustain movement over a range of velocities. The steady rate of oxygen consumption, also known as running economy, is the most common metabolic indicator and represents the sum of various metabolic, cardiorespiratory, biomechanical and neuromuscular characteristics during submaximal running [16, 17].

The preliminary study [15] was extended to include drag considerations and account for the variations in $V{O}_{2}$ for a runner facing wind [18, 19]. The amount of energy expenditure required to overcome air resistance was found to be about 2% for marathon runners [18]. For indoor experiments featuring real runners, extra care should be taken when evaluating the results, since running on a treadmill eliminates wind and air resistance; therefore, conclusions may not apply to overground running [20].

In a 2004 review study, Saunders et al. [21] explored multiple factors, physiological and biomechanical, affecting running economy; however, the effects of aerodynamic drag on running economy were not explored. In a study to investigate the effect of drafting on 3000-m runs, Zouhal et al. [22] raised the possibility that drafting may have a psychological effect in addition to the physiological one.

More recently, different running formations have been adopted, particularly in attempts to break the 2 h barrier over the marathon distance. The first attempt, the Nike Breaking2 project, happened in Monza, Italy in 2017. During the attempt, Eliud Kipchoge and his pacemakers adopted different formations over the course of the race. Although Kipchoge ultimately fell short of the mark at 2:00:25, the attempt was the fastest marathon ever recorded. Despite this remarkable feat, the attempt was not recognized as a world record due to the rotation of the pacemakers, which is not allowed by World Athletics [23].

In a 2018 study, Hoogkamer et al. [24] claimed that a cooperative drafting strategy performed by a team of four elite runners could result in an official marathon time of 2:00:48. Just one month before the publication of the study, at the 2018 Berlin Marathon, Kipchoge adopted different formations as he successively lost his three pacers. Despite the early loss of the pacers, he set a new world record of 2:01:39.

A similar study on cooperative drafting [25] evaluated the running power reduction achieved by Ethiopian runner Kenesisa Bekele, estimating a reduction of metabolic power between 1.91 and 2.84%, depending on Bekele’s position behind his three pacers.

**Fig. 1:**
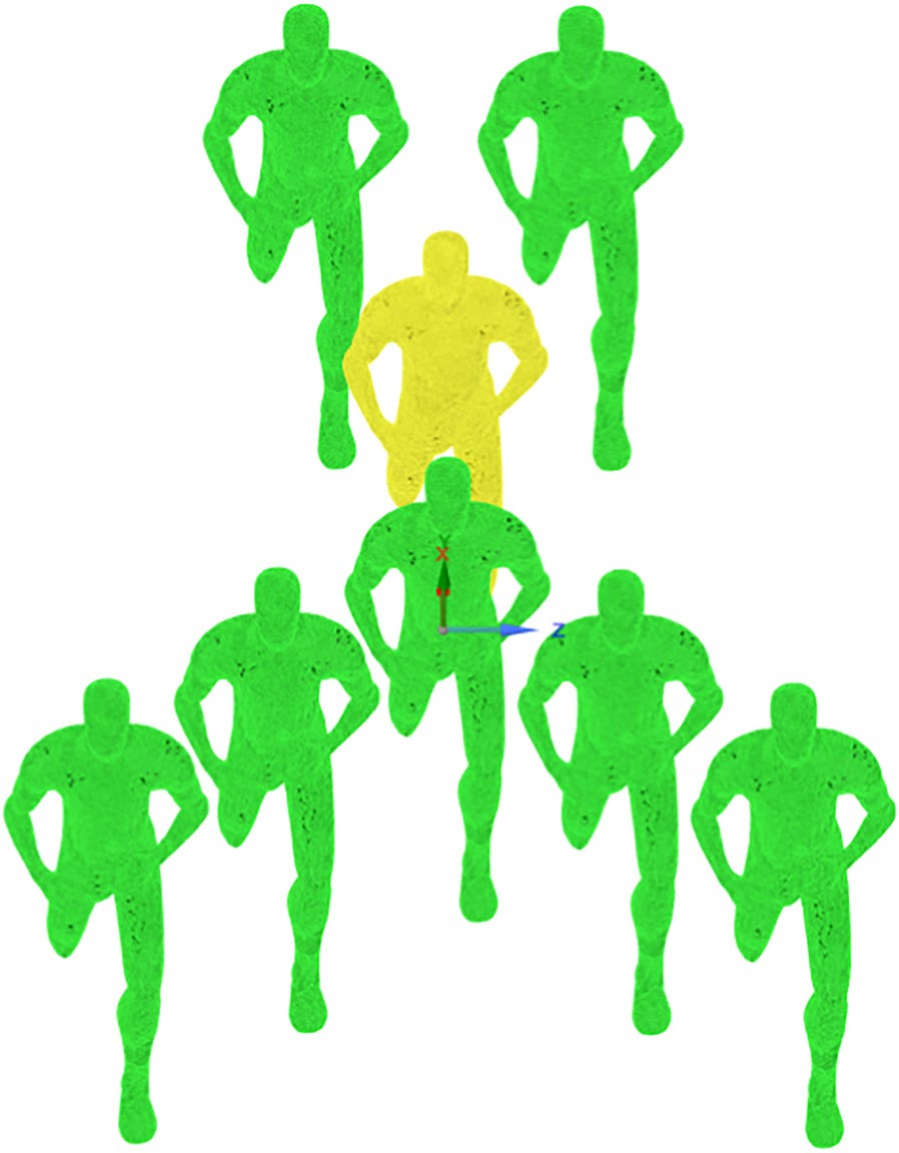
Running formation employed in the 2019 INEOS challenge

In 2019, a second special attempt to break the 2 h mark was made by Kipchoge at the INEOS 1:59 Challenge, in Vienna, Austria. A brand-new formation resembling an inverted arrow (Fig. 1) was employed by Kipchoge (in yellow) and his team of alternating pacers (in green) and he covered the distance in 1:59:40. In a 2022 interview [26], Kipchoge acknowledged the importance of the attempt at Breaking2, despite the goal not being reached; the lessons learned in the race allowed his team to make changes which ultimately led to success in Vienna.

This remarkable achievement has raised curiosity in researchers over the past few years. In a 2020 study, Snyder et al. [27] investigated the effect of curves, elevation and undulations on marathon performance by comparing Kipchoge’s performance in Monza and Vienna; the study concluded that the Vienna course was well chosen when compared to Monza due to fewer curves and elevations, ultimately leading to a 46.24 s advantage.

Recently, several studies have explored marathon running through the perspective of energy to investigate the feasibility of the sub 2 h marathon. The mechanical approach to running is particularly useful since it allows the consideration of possible drag reductions associated with drafting and changes related to pacing strategies.

A 2021 study [28] employed CFD on a realistic 3-D runner model at different speeds to evaluate aerodynamic power and total energy expenditure. Four basic drafting formations were evaluated, and it was found that drag reductions of more than 70% are attainable. However, that study did not include the advanced formations employed by Kipchoge in his fastest marathons (Monza 2017, Berlin 2018, Vienna 2019). Additionally, no previous study considered the possibility of changing formations during the race. For example, in Monza 2017, three formations were employed, even though the pacers were fresh. During the 2018 Berlin marathon, Kipchoge was forced to switch formations three times when his pacers dropped out.

The present study aims to systematically compare different drafting formations to solo running by using a realistic 3-D model of a male runner to perform a CFD methodology. A total of 12 different formations, some previously seen in Kipchoge’s fastest races, were chosen to reflect various scenarios encountered during the attempts. The effect of the drag reduction for each formation on the power output of the runner was calculated using a mechanical power model for running.

Additionally, the mechanical power model and drag values were used to calculate the energy expenditure of each runner during individual races (Berlin 2018 and Berlin 2022). Under the hypothesis of conservation of energy for all runners, the implementation of more effective drafting formations and pacing strategies in different phases of the races leads to improved race times. The consideration of these aspects in an integrated manner allows the optimization of racing strategies, ultimately leading to the sub 2 h official marathon.

## Methods

### Numerical Methods

For the solution of the flow field around the runners and the calculation of corresponding drag values, computational fluid dynamics (CFD) was performed using ANSYS FLUENT. The Navier–Stokes equations were solved in the Reynolds-Averaged (RANS) format.

The geometry for each runner was extracted from a high-fidelity anthropometric model. The model was scaled to properly match Eliud Kipchoge’s height (1.67 m); the limbs were displaced in order to match his running position when the left foot is on the ground, based on publicly available videos.

We chose to perform our simulation using a static model. Previous research for cyclists [29] has shown that leg motion has a minimal impact on instantaneous drag. We assume this is also valid for the flow around a runner. It is important to note that it is indeed possible to perform the simulation considering the movement of the limbs. However, in addition to accurately modeling the movement of each limb, a new mesh has to be created for each time step. This procedure, named dynamic meshing, is usually performed only on very simple geometries, and the computational cost of using it in a very complex geometry is prohibitive.

The turbulence model has an important effect on the solution of the boundary layer, and therefore on the drag force. In this study, the shear-stress-transport (SST) formulation [30] was adopted for turbulence. More information on the geometry and mesh, especially the discretization in the near wall region, is available in “Appendix 1”.

**Fig. 2:**
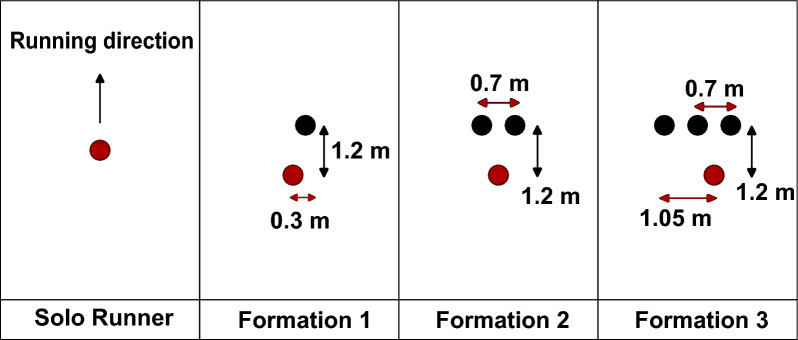
Sketch of the basic formations for the 2018 and 2022 Berlin marathons. Eliud Kipchoge is highlighted in red, and his pacers are depicted in black. In all formations, the direction of forward movement is upward

Eleven different formations, in addition to the solo runner case, were simulated. In both the 2018 and 2022 editions of the Berlin marathon, Kipchoge started the race with three pacers, who dropped out at different moments. The most common running formations for these events are shown in Fig. 2. In each formation, the main runner is represented in red, and the pacers in black. The distance between two runners in the running direction is of 1.2 m, consistent with previous research [25, 28].This distance reduces the risk of tripping.

**Fig. 3:**
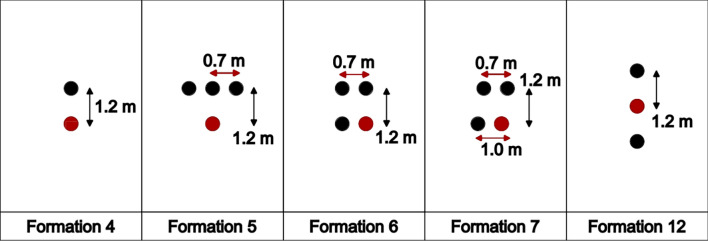
Additional formations related to the 2018 and 2022 Berlin Marathons

We shall highlight a few interesting takeaways about the first three formations, but some additional formations are shown for context in Fig. 3. The lateral distances shown are estimates based on broadcast videos [31–33].

Kipchoge started both races in Formation 3. As long as the three pacers continued, that formation was held at virtually all times. Kipchoge would often change sides within the formation, always positioning himself in a trailing position between two runners. Interestingly, Kipchoge preferred running between two pacers rather than directly behind the center pacer. In rare moments, especially during turns (or at moments when one of the pacers slightly fell below the target pace), some variations for a formation of four runners were seen, such as Formations 6 and 7.

However, these were used for negligible periods of time and are only included in this paper for the sake of completeness. For all racing events, brief changes in formation for hydration stations were disregarded.

**Fig. 4:**
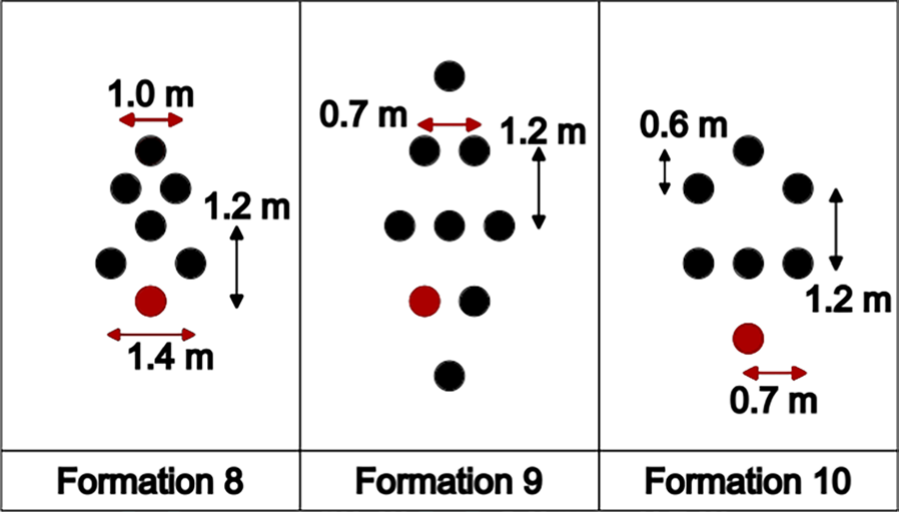
Formations employed during Breaking2 (2017)

The formations adopted during the Breaking2 event (2017) are shown in Fig. 4. Formation 9 was employed from the start of the race until a little over one hour into the race, when two formations of 6 pacers were adopted (Formations 8 and 10). The distances between the runners were estimated after examination of the broadcast [34].

**Fig. 5:**
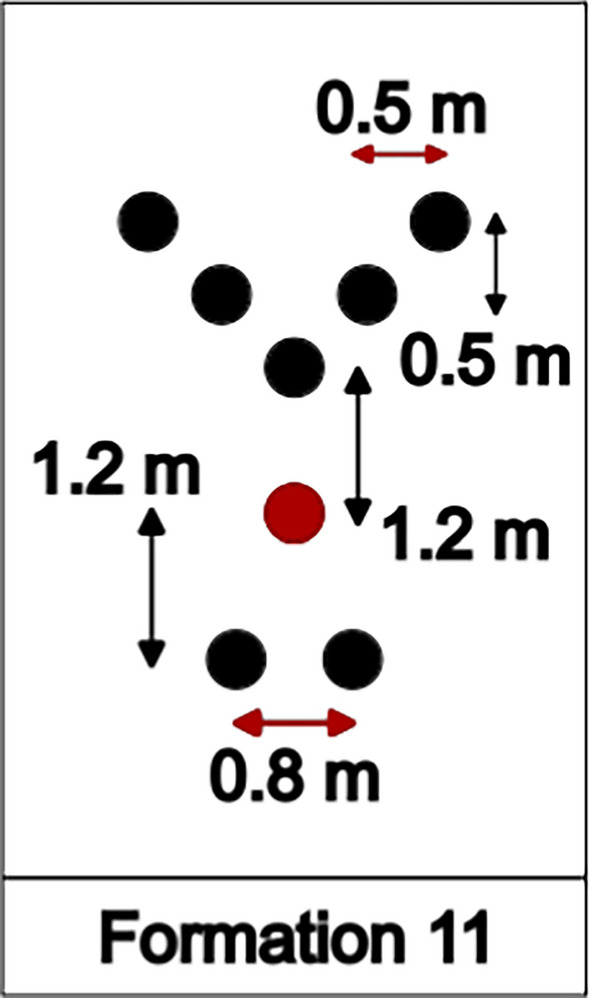
Unique formation employed at the 2019 INEOS challenge

Finally, in the INEOS 1:59 challenge, Formation 11 (shown in Fig. 5) was strictly used during the entire race, save for the instances when the group of pacers was rotated and the final few hundred meters before the finish line. The positions of the five pacers on the ground were projected by lasers located on a pacing car in front of the formation. In the 2021 documentary “Kipchoge—The Last Milestone” [35], a running formation with a distance of 1.5 m in the running direction was shown; however, after careful evaluation of the actual footage of the race [36] and comparison with our CAD model, we found a distance of 1.2 m to be more appropriate.

**Fig. 6:**
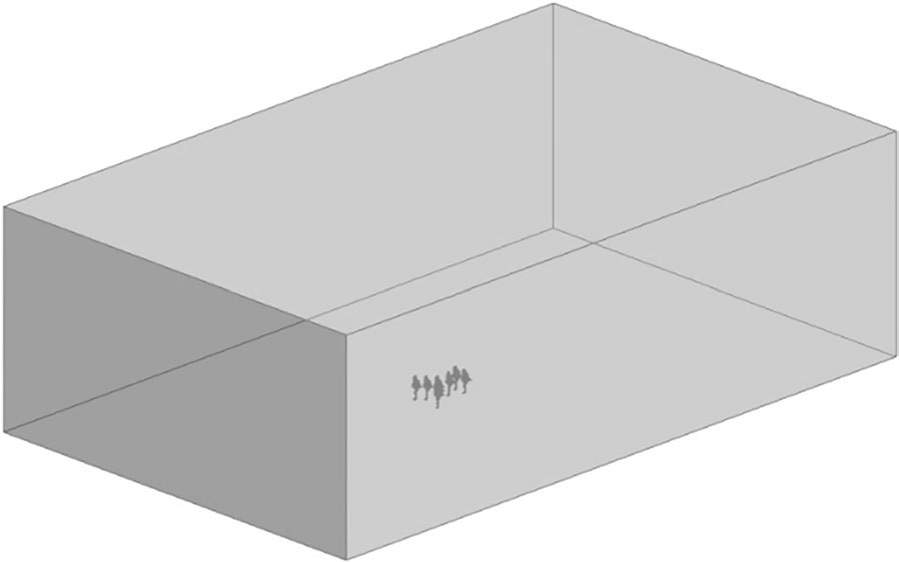
Geometry and fluid domain for Formation 11. For this specific formation, the fluid domain is 85 m long, 50 m wide and 25 m tall. Dimensions vary based on the dimensions of each formation. Our analysis of Formation 11 did not include the pacing car featured at the INEOS challenge

The geometry of the runners for one exemplary Formation (11) is shown in Fig. 6, as well as the surrounding, box-shaped fluid domain. Dimensions for the fluid domain were defined based on best practices for CFD simulations [37]. The lateral and vertical distances between the center of the formation and the walls are defined as twenty-five times the width for each running formation; the axial length is defined as fifty times the length of each formation. Ensuring the boundaries are distant from the object of interest minimizes the effect of boundary condition choices on drag results.

A vertical offset of 2 cm was applied to all the runners, to ensure there is no contact between the lower foot of each runner and the lower boundary. Enforcing all the bodies to be completely surrounded by fluid facilitates the choice of boundary conditions and is indeed a realistic running pose if we consider the instant before the front foot touches the ground.

The inlet boundary condition consists of a uniform velocity of the appropriate value for each case. More information about each case is presented in “Section Methods”. The lateral and upper walls were modeled as symmetric walls, and a static pressure boundary was chosen for the outlet, with an ambient pressure of $p=101325 Pa$. The fluid is defined as air with ambient pressure and a temperature of $20^\circ C$. Although the temperature varied between the different attempts and even within each attempt, the change in air properties is negligible.

The ground is defined as a moving wall, with the same speed as the inlet boundary. This ensures there are no unrealistic boundary layers at the bottom that could invalidate the results. This approach is consistent with previous research [28]. In order to ensure accuracy of the drag values, it is necessary to validate the model. The mesh needs to be fine enough, especially in the near-wall region. A thorough discussion on the validity of the model is present in “Appendix 1”.

### Energetics of Running

In order to compute the power output of a runner, it is necessary to use one of the available empirical models. Perhaps the earliest of such models was proposed by Fukunaga et al. [38], after experiments were performed on athletic runners on strain-gaged force platforms. A relation between running velocity, $u,$ and exerted power for running motion was found as

1 $$P_{R,0} = 0.436 \cdot u^{2.01}$$

With $u$ in $m/s$ and ${P}_{R}$ in $W/kg$. This work was expanded by Cavagna and Kaneko [39], who investigated the additional mechanical power spent to accelerate the limbs relative to the trunk in level walking and running. The more complete equation for the specific running power per unit mass was found as

2 $$P_{R,0} = 9.42 + 4.73 \cdot u + 0.266 \cdot u^{1.993}$$

**Fig. 7:**
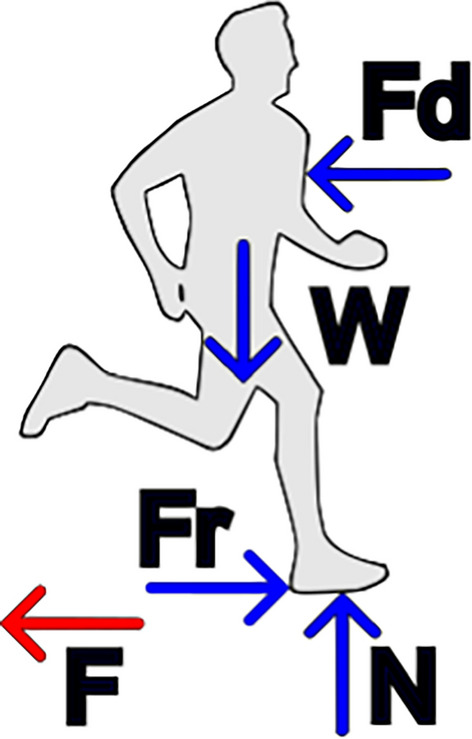
Forces acting on a runner running in straight motion

For this model, the velocity $u$ is defined in $km/h$, and ${P}_{R,0}$ in calories per minute per kg. For the running velocities considered in this study, both approaches yielded similar results, with a maximum difference of 3.06%. At the earliest stages of this study, the model proposed in [39] was used because it includes both the internal and external work performed by a runner. The total mechanical work performed is split into two components:

3 $$W_{tot} = W_{ext} + W_{int}$$

where ${W}_{ext}$ and ${W}_{int}$ are the external and internal components, respectively. The external component accounts for the energy spent to overcome the aerodynamic drag, weight of the runner and ground friction forces (in case one of the feet is in contact with the ground). The forces acting on the body of a runner are shown in Fig. 7. The reaction force to the force $F$, exerted by the feet on the ground, propels the runner forward, according to Newton’s third law. If the runner is running at a steady pace, all the forces acting on his body will be in equilibrium (Newton’s second law).

The internal component accounts for the internal movement of the arms and legs. Past studies have investigated the spring-like motion of muscles during running [40]. More recent studies have also modeled the rotational and translational acceleration of the limbs relative to the trunk [41–43].

Initially, all of the calculations were performed using the model presented in [39]. However, during the review process, a suggestion was made by one of the reviewers to use a more recent (and accurate) model to calculate the running power.

We chose to adopt the model by Tam et al. [44], unmatched in terms of fidelity: the energy cost of running was calculated from experiments performed with elite Kenyan marathon runners in realistic running conditions.

In order to navigate between the different approaches for calculation of mechanical power, a clear distinction must be made between key concepts. Mechanical power is the energy spent to overcome resistance to motion; this involves the forces applied to the ground to propel the body forward and the work done to overcome air resistance and other forces. Metabolic power is the total energy spent by the body to sustain energy; this includes mechanical power and many losses such as heat and the energy used in maintaining bodily functions. Cost of running is the power necessary to move the body over a certain distance; it is converted to mechanical power by multiplication by the velocity.

Since the experiments were performed on a track (and not on a treadmill), the cost of running, ${C}_{r} (\frac{J}{kg\cdot m})$, includes both the internal and external mechanical work components. The total mechanical power, in $W$, is calculated by multiplying ${C}_{r}$ by the velocity and mass of the runner. Modifying the equation for elite Kenyan marathon runners from [44], results in

4 $$W_{tot} = W_{a} + W_{na} = 52 \cdot v \cdot \left( {0.018v^{2} + 3.2833} \right)$$

where ${W}_{a}$ denotes the aerodynamic component of the running power and ${W}_{na}$ denotes the non-aerodynamic power. Usually, models extracted from treadmill data, such as in [39], do not include the aerodynamic power component. The calculation of ${W}_{na}$ from this approach must wait until ${W}_{a}$ has been determined from CFD simulations (“Section Methods”). Once the drag values are determined, the aerodynamic component of running power may be calculated by

5 $${W}_{a}={F}_{D}\cdot v=\frac{1}{2}\rho {u}^{3}{C}_{D}A$$

The drag coefficient, ${C}_{d}$, is a non-dimensional quantity defined to facilitate comparison between different experiments and flow conditions. It is defined below. More information is provided in “Appendix 1”. Full drag results are presented in “Appendix 2”.

6 $${C}_{d}=\frac{{F}_{D}}{\frac{1}{2}\rho {v}^{2}A}$$

The complete evolution of drafting and pacing strategies was performed using the model in [39]; the results are available in “Appendix 3”.

## Analytical Procedure

In order to evaluate Kipchoge’s performance in the four running events in the light of drag benefits generated by drafting, we must develop a procedure that allows for systematic and consistent comparison of different formations and velocities.

As previously mentioned, the formations and velocities change between different races and even within the same race. As a result, the power expenditure also changes. It is, therefore, necessary to split each event into portions that feature one individual formation and to consider the average velocity for each portion. Race breakdowns, for the 2018 and 2022 Berlin marathons are shown in Tables 1 and 2.

**Table 1:** Running splits for the 2018 Berlin Marathon

Time elapsed	Distance covered (km)	*u* (m/s)	Formation
00:41:17	14.23	5.744	3
00:04:22	1.46	5.572	2
00:28:44	9.99	5.794	1
00:47:16	16.515	5.823	Solo
02:01:39	42.195	5.780	–

**Table 2:** Running splits for the 2022 Berlin marathon

Time elapsed	Distance covered (km)	u (m/s)	Formation
01:04:08	22.59	5.870	3
00:06:00	2.07	5.750	1
00:51:01	17.535	5.728	Solo
02:01:09	42.195	5.804	–

In the INEOS challenge, the speed and formation remained constant throughout the attempt. Our goal is to compare Kipchoge’s performance in different races, where applicable. The 2018 and 2022 editions of the Berlin marathon, for instance, are similar; however, the portions of both races where Formation 3 was used were run at different average speeds.

Usually, CFD simulations allow the calculation of drag for one given running speed. With that in mind, the drag values were calculated for four slightly different running speeds for every formation. That process improves the accuracy of our analysis and allows the interpolation of values, which is necessary for our predictions. The lower and upper velocity limits were chosen as the extremes observed in all events, with a lower limit of ${u}_{a}=5.238 m/s$ and an upper limit of ${u}_{d}=5.950 m/s$. The interval was divided into three equal parts, generating intermediate speeds of ${u}_{b}=5.475 m/s$ and ${u}_{c}=5.713 m/s$. Drag values can then be plotted as a function of running speed for all formations.

The drag values calculated from the CFD models are fed into the drag equation and mechanical power model, and finally the power output for each portion of the races is known. Comparison between different races and time savings projections are a complex process, due to varying speeds and formations. However, we use the average running power exerted by the main runner during a certain phase of the run to evaluate possible improvements in case a more aerodynamically effective formation is adopted. Our strategy was developed over several iterations, presented in “Appendix 3”.

It is unrealistic that Kipchoge would have been able to elevate his total energy expenditure during past races, due to a number of factors: hydration, fatigue, sunlight, temperature, among others. Therefore, when speculating that a more effective formation could save him time in a portion of a race, the energy output of the speculated case must match that of the real race.

The procedure of analysis may be summarized as follows:

- Evaluation of broadcasts of the four race events to determine distance covered, time elapsed and formation for each portion of each race;
- Calculation of drag values for CFD simulations for all the formations at the applicable speeds;
- Calculation of aerodynamic, mechanical and total power values;
- Calculation of performance projections based on conservation of energy.

## Results

In this section, the drag results are presented, which are then used to calculate power values and project possible race outcomes. As mentioned previously, our ultimate goal is to show the feasibility of a sub 2 h official marathon. However, early results did not reach that goal (and were calculated using a less accurate model for mechanical power calculation).

The evolution of drafting/pacing strategies consisted of a number of iterations which, despite not achieving the goal, contributed towards the development of our most effective approach. Since the procedure was heavily time-consuming, we chose to keep that portion out of the main paper. If the reader is interested in understanding the evolution of pacing and drafting strategies, all the steps are detailed in “Appendix 3”. In this section, results are presented in simplified form; analysis for the 2018 and 2022 versions of the Berlin Marathon with the finalized drafting/pacing strategy is shown in “Section Optimization problem setup”.

Before getting into race-specific analyses, we shall present the general benefits of drafting.

### Drag Results and Qualitative Analysis

**Fig. 8:**
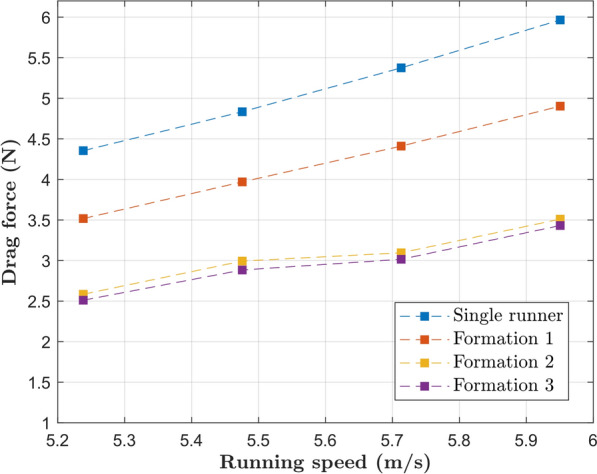
Drag experienced by the main runner for formations used in the 2018 and 2022 editions of the Berlin marathon

**Fig. 9:**
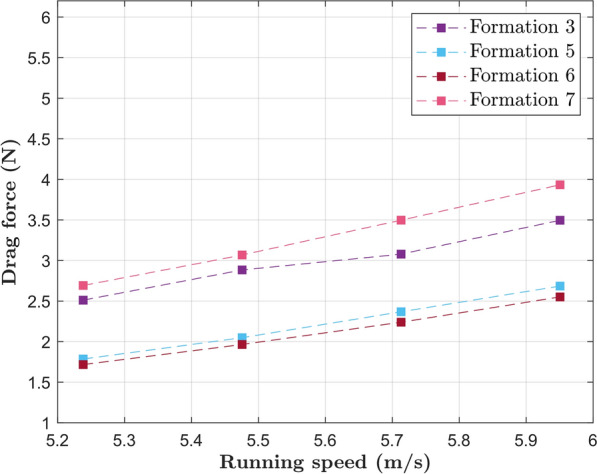
Drag experienced by the main runner in three-pacer formations

**Fig. 10:**
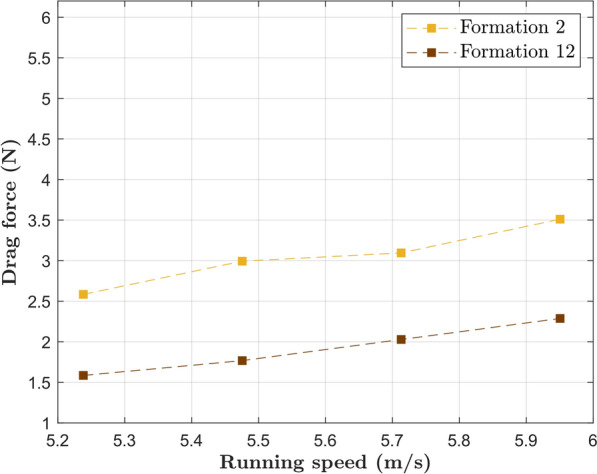
Drag experienced by the main runner in two-pacer formations

**Fig. 11:**
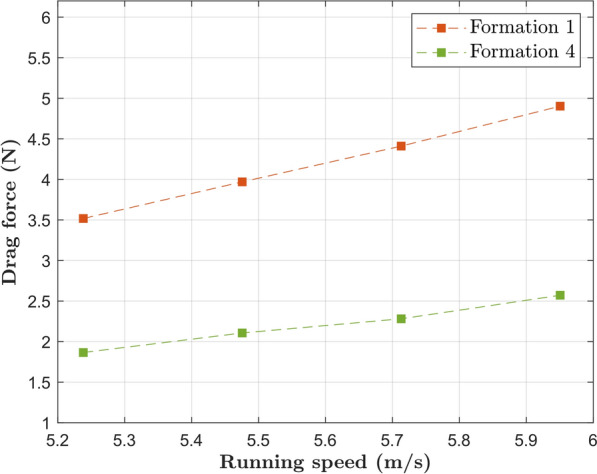
Drag experienced by the main runner in one-pacer formations

In this section, the CFD results for the drag experienced by the main runner in different formations and speeds are presented. Figures 8, 9, 10 and 11 show the pertinent formations for the 2018 and 2022 editions of the Berlin marathon. The advantages of drafting are clearly visible, since the drag experienced by the main runner is significantly higher when running alone than when running behind any number of pacers (Formations 1, 2 and 3). For instance, when running at the target pace of $5.86 m/s$, Kipchoge would experience a drag of $5.73 N$. Running in Formation 1 results in a drag reduction of 19%; Formations 2 and 3 result in drag reductions around 42%.

A comparison between all three-pacer formations is shown in Fig. 9. Formations 5 and 6 are indeed more aerodynamically effective than Formation 3, resulting in drag reductions of around 21% compared to Formation 3 at the target pace. Formation 7 consists of a degeneration of Formation 6 and results in increased drag, resulting in detrimental running performance if used for significant amounts of time.

The drag experienced by the main runner in 2-pacer Formations (2 and 12) is shown in Fig. 10. Formation 12 was a late addition to this study and proved to be the best option when two pacers are available (35% drag reduction compared to Formation 2 at target pace). In fact, it is even more aerodynamically effective than all the three-pacer formations investigated in this study.

Similarly, a comparison between Formations 1 and 4 is shown in Fig. 11. The lateral offset in Formation 1 indeed results in increased drag experienced by the main runner; the adoption of Formation 4 results in a 43% drag reduction at target pace. Comparison between Formations 4 and 12 brings an important observation: the idea of a further drag reduction caused by a pacer running directly behind the main runner is counterintuitive.

The formations employed by Kipchoge and his pacers during the 2018 and 2022 editions of the Berlin marathon were far from being the most aerodynamically effective. In addition to drag values, streamwise velocity plots calculated from CFD simulations help us compare different formations.

**Fig. 12:**
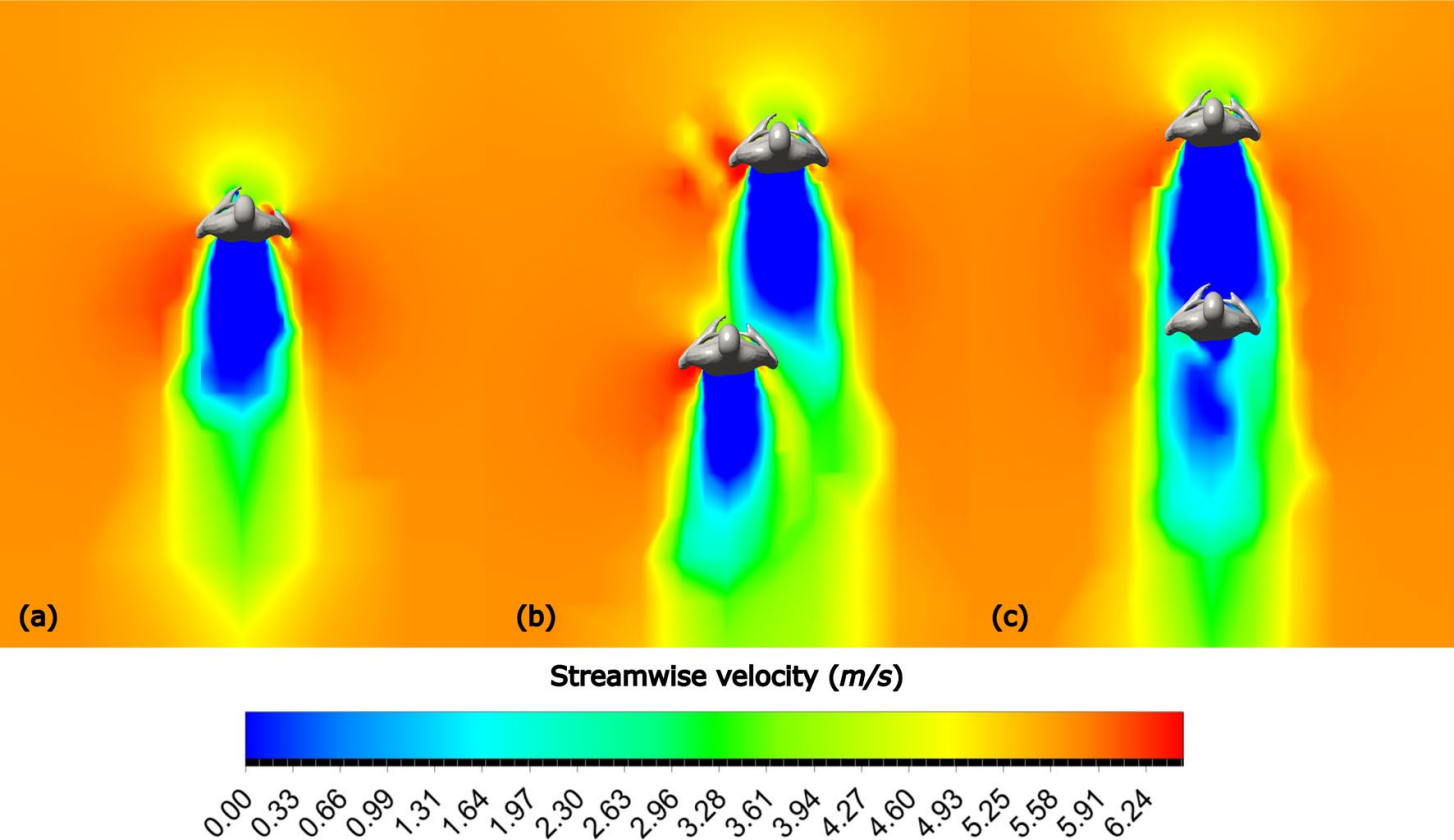
Streamwise contours at $U=5.713 m/s$ for **a** Solo runner, **b** Formation 1, and **c** Formation 4

Streamwise velocity plots for Formations 1 and 4, in addition to the solo runner case, are shown in Fig. 12. When running in Formation 4, the solid blue zone ahead of the main runner represents a lower velocity, resulting in reduced drag; in Formation 1, the green and yellow zones signal increased drag zones.

**Fig. 13:**
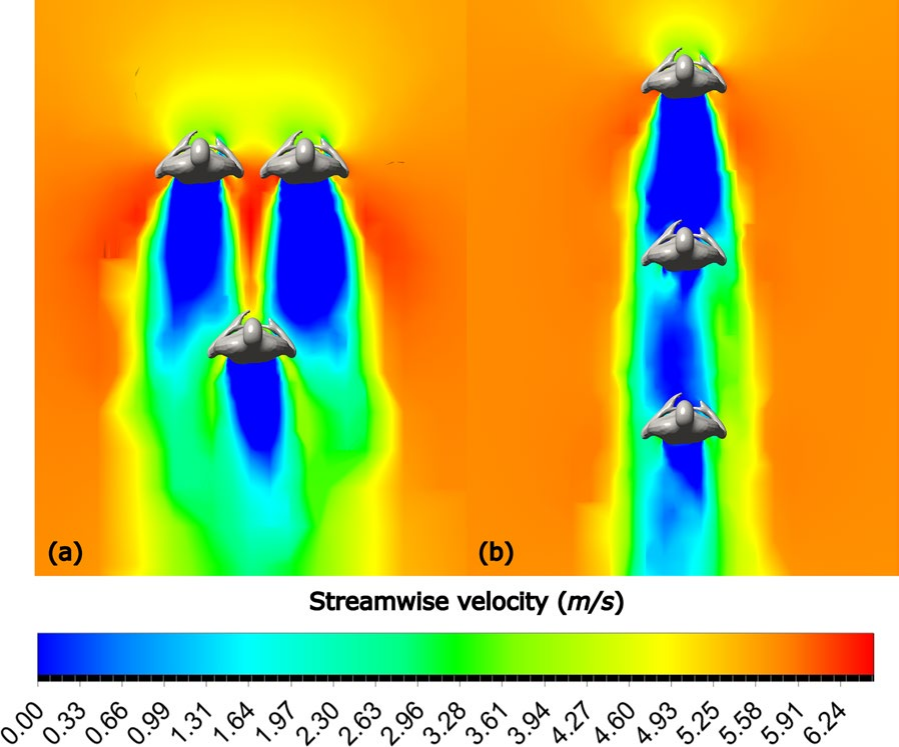
Streamwise velocity contours at $U=5.713 m/s$ for **a** Formation 2, **b** Formation 12

A similar effect is observed for two pacer formations. The streamwise velocity contour plots are shown in Fig. 13. Running in Formation 12 results in a significant stagnation zone ahead of the main runner, in contrast to higher-speed air ahead of the main runner in Formation 2.

**Fig.14:**
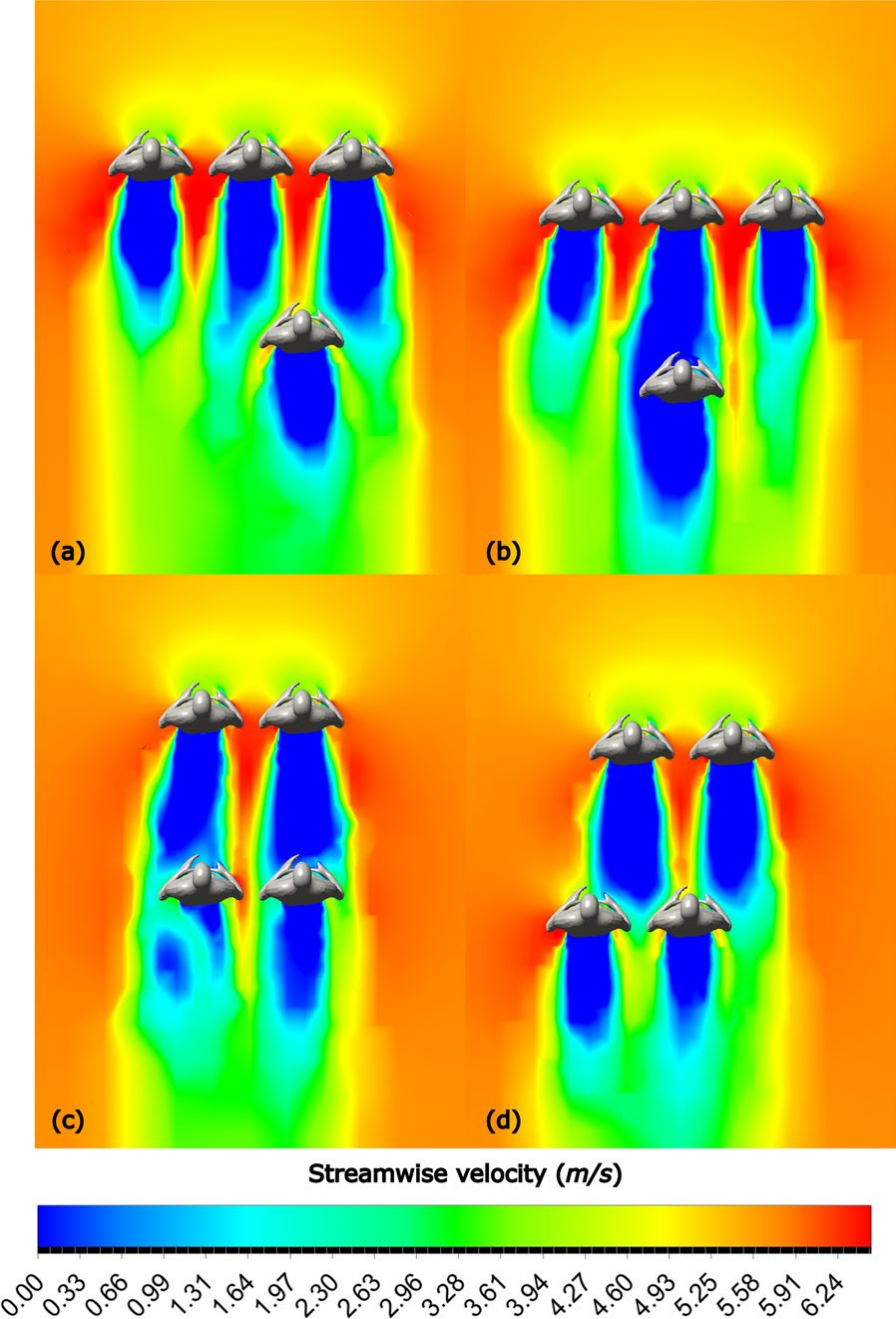
Streamwise velocity contours at $U=5.713 m/s$ for **a** Formation 3, **b** Formation 5, **c** Formation 6, **d** Formation 7

The streamwise velocity contours for three-pacer formations are shown in Fig. 14, highlighting the observations made from the drag plots (Fig. 9).

**Fig.15:**
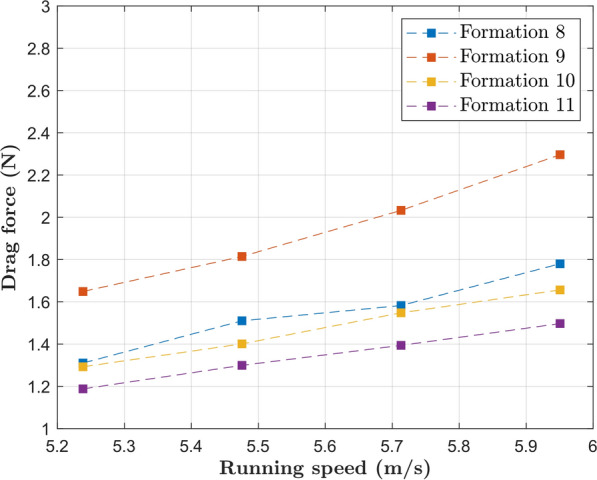
Plots for the drag experienced by the main runner on the formations employed during the two special events

**Fig. 16:**
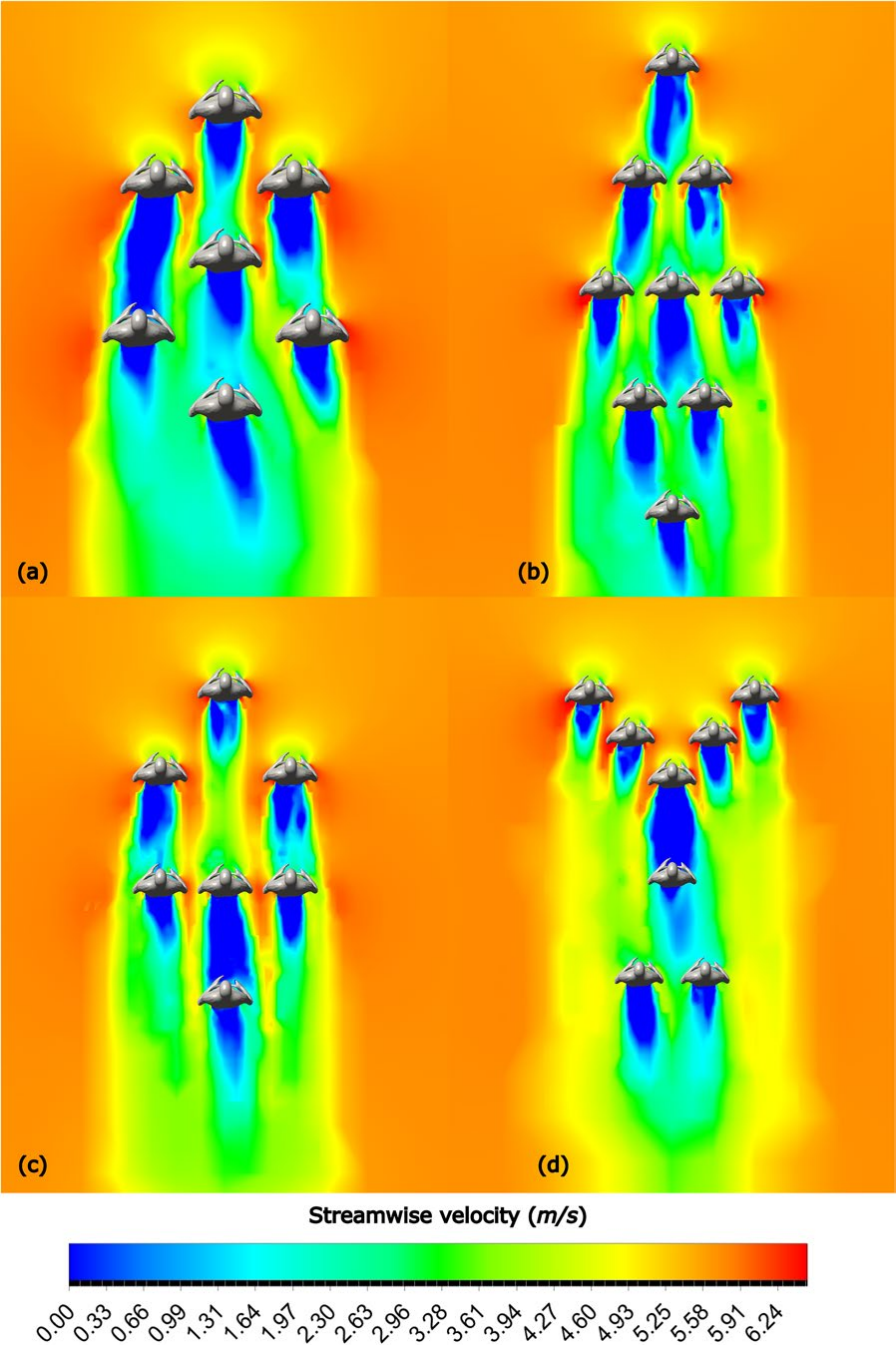
Streamwise velocity contours at $U=5.713 m/s$ for **a** Formation 8, **b** Formation 9, **c** Formation 10, **d** Formation 11

We proceed to the presentation of drag plots for the two special events that were designed to break the 2 h barrier. The drag plots for the formations employed during the Nike Breaking2 and INEOS challenge are shown in Fig. 15. The streamwise velocity contours are shown in Fig. 16.

The experimental nature of the Breaking2 event is evidenced by the different formations employed (8, 9 and 10). The INEOS challenge consisted of Formation 11, indeed the most effective among all investigated in this study, resulting in a substantial drag reduction of 74% when compared to the solo runner case. Formations 8 and 10 also have reduced drag, although not quite as low as Formation 11; perhaps the outlier is Formation 9, that has a higher drag than the alternatives because Kipchoge does not run in the center of the formation.

From these results, one thing is clear: the technical team behind the INEOS challenge did an outstanding job in finding the formation with the lowest possible drag. In Formation 11, Kipchoge runs behind a huge zone of stagnant air, evidenced by the solid blue color in Fig. 16d.

#### Validation of Drag Values

In order to validate our drag values, we turn to extensive existing research estimating the drag experienced by a runner. Direct comparison of drag values is a complicated process due to varying parameters affecting the drag force in different studies (running speed, height and projected area of the runner, surrounding air conditions). For example, a similar CFD study with a runner 1.80 m tall [45] reported drag results 19% higher at target pace.

Fortunately, the drag coefficient, ${C}_{d}$ is useful alternative which considers all of these factors. The drag force, ${C}_{d}$, is assumed to depend only on the Reynolds number as long as there is geometric similitude (i.e., the models have the same shape but different dimensions). Due to different runner poses and projected areas in all CFD and wind tunnel models, it is highly unlikely that ${C}_{d}$ is purely a function of $Re$.

Still, it is useful to compare our results to those established by previous research. Marro et al. [46] compiled the values of ${C}_{d}$ as a function of $Re$ for various research projects featuring both wind tunnel and CFD experiments, all for the solo runner case.

Recent publications by Polidori et al. [25] and Schickhofer [28], both using a CFD methodology like in the current project, reported drag coefficients of $0.81$ and $0.72$, respectively. According to our simulations, Eliud Kipchoge faces about $5.73 N$ of drag when running alone, resulting in a drag coefficient of $0.65$, about 15% lower than lowest reported value.

Results reported from wind tunnel experiments usually report ${C}_{d}$ results within the range of $0.9$ to $1$ [18, 46, 47]. The increased values compared to CFD studies might be caused for two main reasons: firstly, none of the three experimental results reached a close Reynolds number to the realistic one (the highest $Re$ value among the three studies is about 10% of the actual value, about 6.5 million). This is likely due to size and speed limitations of wind tunnel facilities.

Secondly, in wind tunnel experiments, the runner models are fixed on static ground. This generates an unrealistic boundary condition close to the ground, which might be responsible for increased drag values. In the present work, and in accordance with [28] and [25], a moving wall boundary condition was used to deal with that situation, likely leading to more accurate drag values.

It is worth mentioning that the projected frontal area is in line with previous research. Considering similar air conditions, the only parameter affecting the drag coefficient is the drag force. One major aspect is the probable cause for our low drag coefficients: running pose. Research regarding cyclists has reported a drag coefficient of 0.6 for a cyclist in time trial pose and a drag coefficient of 0.8 for the same cyclist in an upright pose [48]. Although the bike will influence the drag coefficient, the difference in pose certainly influences the drag coefficient significantly.

The extent to which running pose affects the drag coefficient can only be evaluated through further research, perhaps using the sliding mesh technique mentioned in “Section Numerical Methods”.

Comparison of drag coefficients for formations (other than the solo runner case) are not possible due to differences in formation geometries among different studies. Perhaps the most useful comparisons are drawn between the drag reduction associated with drafting (as a percentage of the drag faced by a solo runner).

As one example, Schickhofer [28] reported drag reductions of 70.1%, 75.6% and 41.3% for Formations 4, 12 and 2, respectively, when compared to the solo runner case at $5.83 m/s$. Our results for the same formations indicate drag reductions of 54%, 62% and 42%, respectively. However, differences in running pose and distances within the formations likely contribute to these discrepancies.

Finally, it is appropriate to mention that, in the documentary “Kipchoge – the last milestone” [35], the drag experienced by Kipchoge was reported to be about $6 N$. It is unclear whether these results were calculated from real racing experiments, CFD or wind tunnel experiments. However, the documentary briefly shows wind tunnel experiments which contributed to the development of Formation 11.

### Calculation of Power Values

The next step in our analysis consists of calculating the total running power for all pertinent formations and velocity values. Second order polynomial fits for the drag values were calculated for all runners in pertinent formations from CFD simulations; the results are shown in Table 9 (“Appendix 2”).

The calculation of aerodynamic power for the solo runner case finally allows us to determine the mechanical running power using the equations provided in “Section Energetics of Running”. The non aerodynamic running power is calculated as

7 $${W}_{na}=52\cdot v\cdot \left(0.018{v}^{2}+3.2833\right)-v\cdot \left(0.493{v}^{2}-3.256v+7.878\right)$$

The first portion represents the total running power calculated from the experiments in [44]. After subtracting the second portion, i.e. the aerodynamic power, the portion equivalent to the mechanical power in the model by Cavagna and Kaneko [39] is calculated.

The running power for the appropriate running formation and position are added to ${W}_{na}$ in order to accurately calculate the energy expenditure of each runner during each phase of the races. The running splits for the 2018 and 2022 editions of the Berlin marathon are presented in Tables 1 and 2.

The application of the running power equations for the pertinent formations and velocities allows for the calculation of the total energy expenditure of each runner during the two races. The results are summarized in Tables 3 and 4. For reasons discussed in “Appendix 3”, only the two best pacers matter to our analysis.

**Table 3:** Energy expenditure for each runner in the 2018 Berlin Marathon

Runner	Energy expenditure (10^6^ J)
Eliud Kipchoge	9.1535
Josphat Boit	5.6024
Sammy Kitwara	3.424

**Table 4:** Energy expenditure for each runner in the 2022 Berlin Marathon

Runner	Energy expenditure (10^6^ J)
Eliud Kipchoge	9.1546
Jacob Kiplimo	5.4148
Isiah Koech	4.9674

### Conservation of Energy and Performance Projections

Once the energy expenditure for each runner is known, it is possible to project alternative race outcomes based the adoption of different drafting and pacing strategies. Once again, the interested reader may find the complete evolution of our drafting and pacing strategies in “Appendix 3”. Here, we briefly list the drafting and pacing guidelines for optimal performance in official marathon events:.

**Fig. 17:**
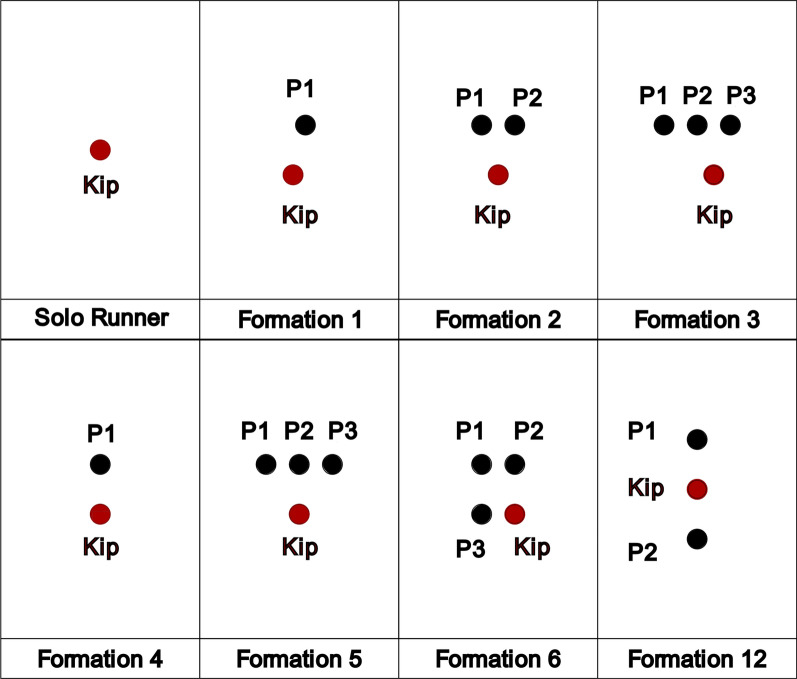
Pacer numbers for appropriate formations

- Prior to the start of the race, determine the two strongest pacers, who, along with Kipchoge, start the race in Formation 12.
- Start the race running at velocity ${v}_{1}$. The strongest pacer occupies the P1 position (Fig. 17, “Appendix 2”). The weakest pacer occupies position P2. Run in Formation 12 until the weakest pacer extinguishes his energy and drops out.
- As soon as the weakest pacer drops out, switch to Formation 4, with the remaining pacer in front of Kipchoge. Run in velocity ${v}_{2}$ until the pacer extinguishes his available energy and drops out.
- Kipchoge runs the last portion of the race alone at velocity ${v}_{3}$.

Ideally, the optimal pacing strategy will minimize total race time while making sure all three runners use all of their available energy. Our experience from “Appendix 3” advocates for the use of a positive splitting strategy (i.e. starting the race faster and reducing the speed once pacers drop out, ${v}_{1}>{v}_{2}{>v}_{3}$), our final strategy consists of keeping ${v}_{1}, {v}_{2}$ and ${v}_{3}$ as variables and setting up an optimization problem. The optimal results will confirm if a positive splitting strategy is better indeed.

### Optimization Problem Setup

Here, the outline of the optimization algorithm is presented. These schemes were implemented in MATLAB, and the scripts are available in the Supplementary materials. A standard interior-point method, implemented through function *fmincon,* was used for its fast convergence and adequacy for non-linear, constrained optimization problems like ours [49].

**Table:** **Algorithm 1** Calculation of optimal race time

Definition of maximum energy values for each runner;	
Declaration of variables for running velocities ${v}_{1}, {v}_{2}$ and ${v}_{3}$;	
**Calculations for first phase of the race (Formation 12, as functions of ** ${{\varvec{v}}}_{1}$ **)**	
Calculation of power expenditure by the weaker (limiting) pacer, P2, Formation 1;	
Time in phase 1, ${t}_{1}$, calculated by dividing the available energy by instantaneous power of weaker pacer;	
Calculation of energy expenditure by Kipchoge and stronger pacer;	
Calculation of distance covered in phase 1, ${v}_{1}{t}_{1}$;	
Update on the remaining available energy for Kipchoge and stronger pacer	
**Calculations for second phase of the race (Formation 4, as functions of ** ${{\varvec{v}}}_{2}$ **):**	
Calculation of power expenditure by the stronger (now limiting) pacer, P1, Formation 4;	
Time in phase 2, ${t}_{2}$, calculated by dividing the available energy by instantaneous power of stronger pacer;	
Calculation of energy expenditure by Kipchoge; update on the remaining energy available	
Calculation of distance covered in phase 2, ${v}_{2}{t}_{2}$;	
**Calculations for third phase of the race (Solo runner, as a function of ** ${{\varvec{v}}}_{3}$ **):**	
Calculation of remaining race distance:$42195-{v}_{1}{t}_{1}-{v}_{2}{t}_{2}$	
Calculation of time spent in phase 3:${t}_{3}=\left(42195-{v}_{1}{t}_{1}-{v}_{2}{t}_{2}\right)/{v}_{3}$	
Calculation of energy spent by Kipchoge in phase 3	
**Optimization problem setup:**	
Objective function defined as $f={t}_{1}+{t}_{2}+{t}_{3}$;	
Constraint for the velocities: ${v}_{1},{v}_{2},{v}_{3} \in \left[\text{5,6}\right] m/s$;	
Energy constraint for Kipchoge: remaining energy equals energy spent in phase 3;	
Run optimization algorithm and gather results	

### Optimization Results

The optimization algorithm was employed to simulate results for both the 2018 and 2022 editions of the Berlin marathon, predicting a possible sub 2 h finish in both races. The results are shown for the 2018 Berlin marathon in Table 5, predicting a finish of 1:59:53 (rounded up to the next second)—1 min and 46 s faster than the actual race.

**Table 5:** Optimization results for the 2018 Berlin marathon

Berlin 2018			
Phase	Speed (m/s)	Distance (m)	Time (s)
1	5.967	15,800.68	2647.70
2	5.923	9717.44	1640.60
3	5.743	16,676.81	2903.80
Total	5.8668	42,195	7193

Table 6 shows the pertinent results for the 2022 Berlin marathon, predicting a 1:59:48 finish—1 min and 21 s faster than the actual race.

**Table 6:** Optimization results for the 2022 Berlin marathon

Berlin 2022			
Phase	Speed (m/s)	Distance (m)	Time (s)
1	5.967	22,919.34	3840.50
2	5.923	1734.61	292.85
3	5.743	17,541.46	3054.30
Total	5.870	42,195	7188

## Discussion

### On the Method of Conservation of Energy

The conservation of energy for each runner between the actual and projected race must be considered carefully. The limiting factor for marathon performance is not the total energy available to each runner, but rather for how long he can sustain a certain power output.

A possible improvement to our work is available through a change in the restrictions of the optimization problem: rather than enforcing conservation of energy directly, it is possible to restrict the power output of each runner in time during the simulated race. The limiting value for each time is the power output for that runner during the actual race. In doing so, the total energy spent during the simulated race would automatically be lower or equal to that of the real race, satisfying our energy constraints.

Also, in the current project, a constant metabolic efficiency was assumed at all running speeds; this assumption is questionable. Other methods, such as the one proposed in [50], are backed by empirical measurements and therefore present a more accurate way of capturing nonlinear velocity effects on the cost of running.

Overall, this work contains many simplifying assumptions; for example, all calculations are averaged, i.e., instant fluctuations in power output (caused by slight acceleration/deceleration, formation changes, etc.) are not considered. It is difficult to account for the error caused by conservation of energy and the assumption of a constant metabolic efficiency. Ultimately, these topics are beyond the scope of this paper.

### On the Feasibility of the Sub 2 h Marathon

The optimization results make it clear that improved drafting/pacing strategies can indeed help Eliud Kipchoge (or other elite runners) break the two-hour barrier for official marathons. The implications of the current results are major in the field of sports science. However, a few limitations must be highlighted.

Although the geometry employed in our CFD simulations was generated from a high-fidelity human model, the running positions, as well as the dimensions in the formations, are estimates. In real races, changes in these factors will affect the drag. In this project, we assume this does not affect the results to a significant extent.

Our models also do not consider curves and elevation changes in any of the courses analyzed. A complete study on the subject [27] showed that these factors may result in increased racing times compared to a perfectly flat and straight course. The inclusion of these aspects would require a total shift in the scope of this paper. Additionally, since the course does not change between the actual and projected races, it is likely that the influence (if any) of curves and elevations in our results is minimal.

One important aspect of the calculation of non-aerodynamic power ${W}_{na}$ is of interest. In [44], data from the elite Kenyan runners was gathered at high altitude. Due to decreased oxygen availability, running power tends to be higher in these conditions. It is likely that the same runners would have a reduced running cost had the data been extracted at sea level.

The use of equations based on high-altitude running in our analysis is a conservative factor. Lower, more realistic running power values would mean increased significance of the aerodynamic power (the portion of power which can be effectively controlled by drafting). The changes in the drag experienced by a runner between sea level and Kenya are somewhat significant due to the change in air properties; however, since metabolic power accounts for 96–98% of the total running power, we assume metabolic effects due to high altitude to be dominant.

Ultimately, the race projections require that each runner develops a certain power output during different phases of the race. Other than conserving the total energy, our model does not predict whether each runner would be able to output the required power as dictated by the optimization algorithm. This can only be achieved through the consideration of higher-level biomechanical aspects.

### On Positive Splitting

The optimization results indeed show that a positive splitting strategy is preferable in terms of total race times, as predicted by the evolution of our pacing strategies in “Appendix 3” and contrary to popular belief that negative splitting strategies are a better choice. Our optimization results require that phase 3 (when no pacers are present) be run at speeds 3–4% lower than phases 1 and 2.

The extent to which positive splitting is the better strategy is limited to races when two pacers are available for significant time. This subject has been investigated by past research [51]. In a simplified manner, the dilemma faced by an elite runner when choosing the pacing strategy is defined by two contradictory options:

- A negative splitting strategy starts the race slower, preserving energy for both the main runner and the pacers. The pacers stay in the race for longer, affording the main runner drag benefits for extended time. Finishing the race faster means that the main runner experiences very high drag towards the end, thus requiring a very high power output.
- A positive splitting strategy starts the race faster, depleting energy for both the main runner and the pacers faster (and thus keeping the pacers in the race for less time). The increased start speed means that speed may be reduced towards the end (when the main runner does not experience any drafting advantages), requiring a lower power output to finish the race.

Interestingly enough, in Kipchoge’s fastest actual race (2022 Berlin Marathon), a positive splitting strategy was used (as evidenced by Table 2) and contrary to the negative splitting strategy used in the 2018 Berlin marathon (Table 1). This might be the result of research done by his team and certainly helped him achieve his personal best (and then world record) on that occasion.

### On the Possibility of Cooperative Drafting

The idea of cooperative drafting (i.e., alternating the position of pacers to keep them in the race for longer) has been suggested in past research [24, 25] as a means to improve race times. Although not the focus of this research project, the authors decided to investigate the effect of alternating pacers in phase 1 (Formation 12), under the assumption that it is possible to split the time spent in phase 1 exactly in half.

The adoption of cooperative drafting under these specific circumstances resulted in a further time savings of 1.21 s for the 2022 Berlin marathon and 0.75 s for the 2018 Berlin marathon. These time savings are negligible compared to those associated with standard drafting and pacing.

Additionally, given the hurdles associated with cooperative drafting, such as definition of shifts and changes of formations (resulting in increased risk of tripping), it is the opinion of the authors that cooperative drafting should be avoided for official marathon events. Also, the aforementioned studies considered a group of four equally strong runners (not a designated runner and his pacers). That assumption does not translate fully into the current paper.

### On the Simulated Performance Improvement for the 2018 and 2022 Berlin Marathons

The optimization results for the 2022 and 2018 editions of the Berlin marathon are interesting in the sense that the race times are very close (and the velocity values for each phase are virtually the same). Why did the optimization result in higher time savings for 2018 (106 s) compared to 2022 (81 s)?

The answer to this question is based on two aspects, the first of them already mentioned: the positive splitting adopted in the 2022 Berlin marathon left less room for improvement compared to the negative splitting strategy adopted in the 2018 event.

The second aspect is related to the availability of pacers. In 2018, the weakest pacer stayed in the race far less than in 2022; however, the strongest pacer remained for longer than in 2022. When projecting races based on alternative drafting strategies, it is likely more beneficial to have a solo pacer for a longer time than two simultaneous pacers for a shorter time.

### On the Limitations of Static CFD Simulations

One limitation of CFD simulations performed in a static domain is the inability to fully capture the effects of flow transition, which can introduce non-linearities between the drag coefficient and Reynolds number. As noted by Crouch et al. [29], the Reynolds numbers relevant to limbs at race speeds are often near the drag crisis experienced by a circular cylinder. This suggests that flow transition may significantly impact drag in ways not fully represented by static simulations.

A further indication of these effects is highlighted by the fact that Kipchoge wore tape strips with vortex generators on his calves on both the 2018 edition of the Berlin Marathon.

## Conclusion

In the present work, multiple drafting formations were systematically compared with respect to drag and mechanical power output for a marathon runner. In an attempt to show that Kenyan runner Eliud Kipchoge can run an official marathon event in under two hours, several drafting/pacing strategies were developed, culminating in a strategy that could have allowed Kipchoge to run the 2022 Berlin marathon in 1:59:48 and the 2018 Berlin marathon in 1:59:53. Both optimized races feature positive splitting pacing strategies, contrary to popular belief in the superiority of negative splitting. Our results present important information for elite marathon runners aiming to eventually break the two hour mark in official events.

## Figures and Tables

**Fig. 18:**
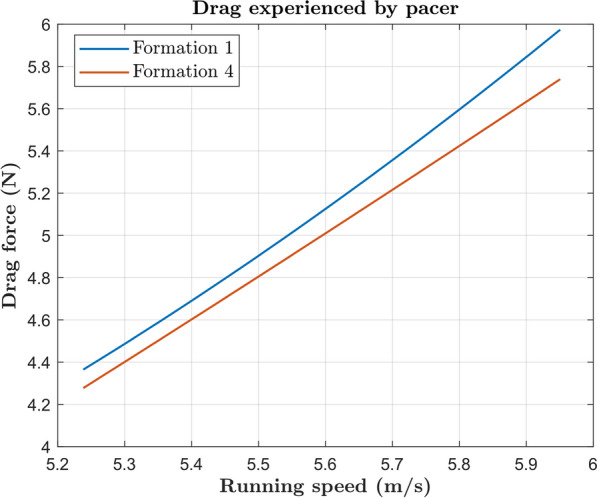
Drag experienced by sole pacer in Formations 1 and 4

**Fig. 19:**
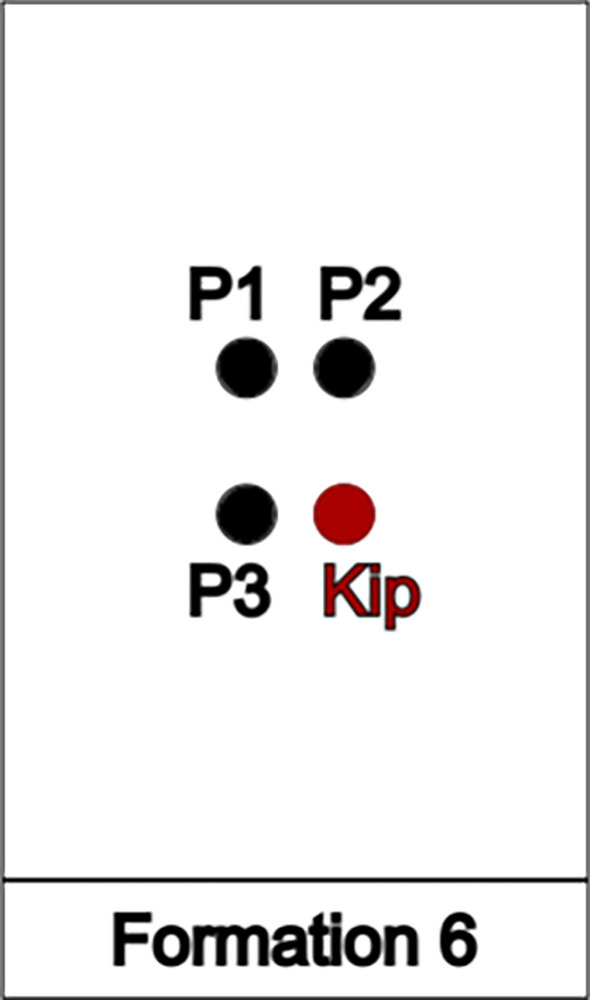
Detailed view of Formation 6

**Fig. 20:**
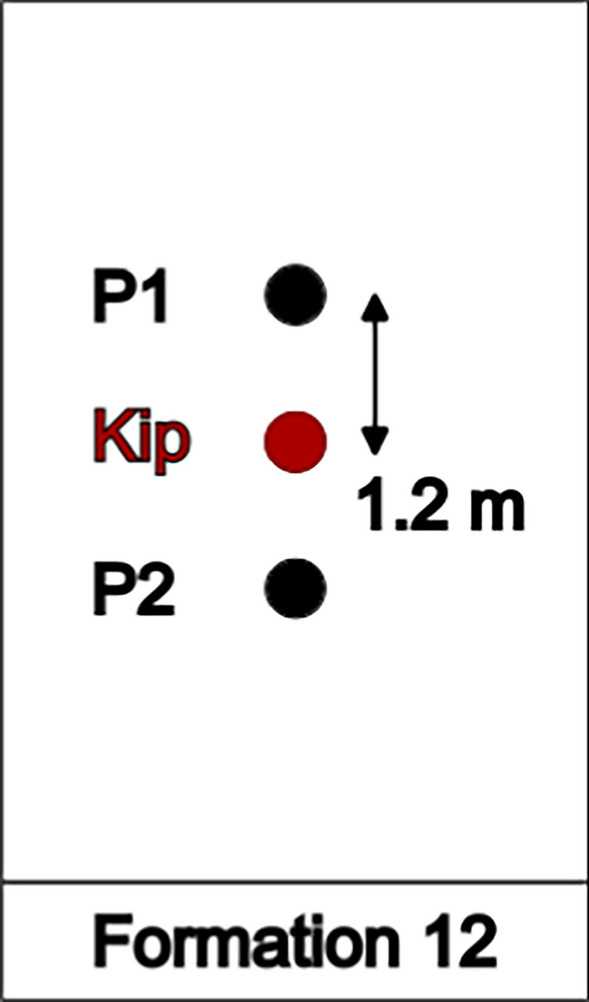
Detailed view of Formation 12

**Fig. 21:**
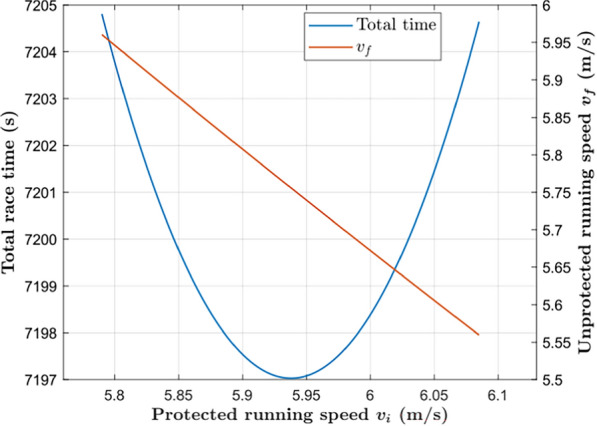
Projected unprotected speed and total race time for the 2022 Berlin marathon

**Fig. 22:**
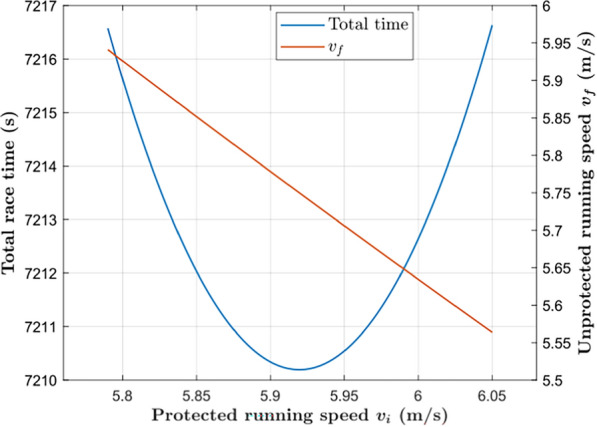
Projected unprotected speed and total race time for the 2018 Berlin marathon

## Data Availability

All the relevant material is included either in the manuscript or in the appendix.

## Appendix 1

### Additional CFD Information

#### Geometry and Mesh

A very important remark must be made about the 3-D models employed in the CFD simulations. We chose to use a static geometry that represents one frame of the runner’s movement for simplification purposes. There are multiple reasons why this is a valid alternative; first of all, it was shown in previous research that leg movement has a negligible influence on the drag experienced by cyclists [29]. Although no studies have been performed investigating if that also applies to running, we assume this also applies to our study.

The second reason is the fact that accurately modeling the motion of arms and legs, as well as the core, would require extensive body measurements and other experiments performed on the runner.

Finally, the application of CFD on a moving geometry would require a sliding mesh technique (i.e., regenerating the mesh at very small intervals when the position of the limbs is altered). This process, although the most accurate approach, has a prohibitive computational cost, especially for such a complex geometry with many moving parts.

We used the same 3-D model for the main runner and all the pacers; the 3-D model was scaled to match Eliud Kipchoge’s height of 1.67 m.

It is necessary to know the runner’s projected area to calculate the drag coefficient ${C}_{d}$ and aerodynamic power ${P}_{A}$. The first alternative to estimate $A$ is by using the following relation [52]:

8 $$A=0.0597\cdot {h}^{0.725}\cdot {m}^{0.425}$$

where $h$ is the height in meters and $m$ the mass in kilograms. Publicly available information about Eliud Kipchoge [53] lists his height as 1.67 m and his weight as 52 kg; the projected area is then estimated as 0.464 m^2^. The second alternative is provided by SOLIDWORKS, which automatically informs a projected area of 0.43 m^2^. There is good agreement between the two, but we ultimately decided to use the latter since it is a more accurate description of our model.

The mesh was generated using the watertight geometry workflow in ANSYS FLUENT, which has a dedicated meshing application. The minimum element size was chosen as 0.002 m, in order to generate a minimum discretization of 100 elements in the streamwise direction. The domain dimensions are chosen so that the flow is undisturbed close to the bodies and there is little influence caused by specific boundary conditions. Other meshing sizing parameters were chosen in accordance with best practices [37].

Finer discretization is necessary close to the surface of the runner’s body in order to accurately model the boundary layer. The chosen turbulence model is the SST model [30], for which a ${y}^{+}$ value of less than 1 is desirable. A 30-layer inflation was applied on all bodies, with the first element height of $5.84 {10}^{-5} m$, estimated through the method detailed below.

### Reynolds Number

The Reynolds number (Re) is a dimensionless quantity that helps predict fluid flow patterns in different situations. For example, fluid properties, such as density and viscosity may be different; flow velocity may also be altered, as well as the geometric dimensions of the object immersed in the fluid. The Reynolds Number is defined as

9 $$Re=\frac{\rho \cdot {u}_{\infty }\cdot d}{\mu }$$

where $\rho$ is the fluid density, $u$ is the flow velocity, $d$ is a characteristic dimension and $\mu$ is the dynamic viscosity of the fluid. For a runner, the characteristic dimension, $d$, is usually chosen as the runner’s height. Considering a height of 1.67m and a running speed of $5.86 m/s$, the Reynolds number is calculated as

10 $$Re=\frac{1.204\cdot 5.86\cdot 1.67}{1.831\cdot {10}^{-6}}=6.43\cdot {10}^{5}$$

The significance of Re for a runner is in the fact that the drag coefficient, ${C}_{d}$, is a function of it; More generally, it is important especially for wind tunnel experiments, when scaled models are used; in that case, if the Reynolds Numbers for real life and experiment are matched, values for ${C}_{d}$ will be the same.

### Wall Treatment and Boundary Layer

There are two options to approach the near wall region in RANS simulations: the use of wall functions or a proper solution for the viscous sublayer, the choice for this study. For low-speed subsonic conditions, this region must be adequately discretized in order to calculate drag with good accuracy [37]. The turbulence model chosen is the SST model [30], which imposes a condition in the dimensionless wall distance value over the geometry of ${y}^{+}<3$.

The actual values of ${y}^{+}$ are only known after the simulation is completed. Therefore, it is necessary to make an initial estimate so that proper discretization of the near wall region is possible. In our study, we chose to use an inflation consisting in 30 prismatic layers with a growth factor of 1.2. The height of the first layer, $\Delta s$, is calculated through appropriate equations [2], also shown below:

11 $${C}_{f}=\frac{0.026}{R{e}^{1/7}}$$

12 $${\tau }_{w}=\frac{{C}_{f}\rho {U}_{\infty }^{2}}{2}$$

13 $$\Delta s=\frac{{y}^{+}\mu }{\sqrt{\frac{{\tau }_{w}}{\rho }}\rho }$$

The Reynolds number has been previously calculated as $6.51\cdot {10}^{5}$; the air density is, once again, equal to $1.219 kg/{m}^{3}$. The dimensionless wall distance ${y}^{+}$, was conservatively chosen as $1.0$, well inside the required threshold ${y}^{+}<3$ [30, 37].

## Appendix 2

### Drag Results and Second-Degree Polynomial Fits

See Fig. 17 and Tables 7, 8, 9, 10, 11 and 12.

**Table 7:** Drag reduction experienced by the designated runner at $U= 5.95\frac{m}{s}$

Formation	Drag (N)	Reduction (%)
Solo	5.9652	–
1	4.8787	18.21
4	2.8298	52.56
2	3.4715	41.80
12	2.2871	61.66
3	3.4961	41.39
6	2.465	58.68
5	2.3684	60.30
7	3.4974	41.37
8	1.5524	73.98
9	2.0326	65.93
10	1.5479	74.05
11	1.4229	76.14

**Table 8:** Drag values (N) for all runners and formations

Velocity (m/s)		5.2381	5.4756	5.7131	5.9506
Formation	Runner				
Solo	Kipchoge	4.3545	4.8335	5.3749	5.9652
1	Kipchoge	3.4779	3.8962	4.3726	4.8787
	P1	4.3512	4.8913	5.3467	5.9873
4	Kipchoge	1.9118	2.1854	2.4982	2.8298
	P1	4.2736	4.7667	5.2320	5.7431
2	Kipchoge	2.5479	2.9359	3.0549	3.4715
	P1	4.7176	5.2740	5.5962	6.1914
	P2	4.7786	5.3104	5.6424	6.2036
12	Kipchoge	1.5854	1.7680	2.0287	2.2871
	P1	4.1850	4.5907	5.0067	5.5428
	P2	1.8629	2.1054	2.3758	2.6390
3	Kipchoge	2.4168	2.6843	3.0784	3.4961
	P1	5.0661	5.4858	6.1088	6.7267
	P2	5.1293	5.5760	6.2322	6.8808
	P3	4.7908	5.1842	5.8945	6.4642
6	Kipchoge	1.8313	2.1309	2.4650	2.8090
	P1	4.5546	5.0920	5.6072	6.2030
	P2	4.6279	5.1493	5.6739	6.2494
	P3	2.0352	2.3125	2.6257	2.9714
5	Kipchoge	1.7860	2.0485	2.3684	2.6846
7	Kipchoge	2.6918	3.0678	3.4974	3.9329
8	Kipchoge	1.3102	1.5100	1.5524	1.7800
9	Kipchoge	1.6486	1.8146	2.0326	2.2960
10	Kipchoge	1.2924	1.4010	1.5479	1.6561
11	Kipchoge	1.1880	1.2992	1.4229	1.4969

**Table 9:** Second order polynomial fit coefficients for drag values, $a{u}^{2}+bu+c$, for select formations

Coefficient		a	b	c
Formation	Runner			
Solo	Kipchoge	0.4933	−3.2568	7.8784
1	Kipchoge	0.3891	−2.3840	5.2868
	P1	0.4454	−2.7254	6.4189
4	Kipchoge	0.2571	−1.5849	3.1595
	P1	0.0798	1.1595	−3.9853
2	Kipchoge	0.1268	−0.2015	0.1538
	P1	0.1720	0.0732	−0.3589
	P2	0.1303	0.4819	−1.2992
12	Kipchoge	0.3360	−2.7628	6.8352
	P1	0.5780	−4.5762	12.3040
	P2	0.0918	0.0674	−1.0098
3	Kipchoge	0.6657	−5.9191	15.1510
	P1	0.8784	−7.4688	20.0750
	P2	0.8948	−7.5235	19.9750
	P3	0.7814	−6.3298	16.4850
6	Kipchoge	0.1968	−0.8261	0.7580
	P1	0.2588	−0.5969	0.5846
	P2	0.2398	−0.4137	0.2184
	P3	0.3032	−2.0775	4.5992

**Table 10:** Power values for Solo runner and one-pacer formations (1 and 4)

			Single	Formation 1		Formation 4	
Interval	U	PR	Kip	Kip	PI	Kip	PI
1	5.2381	693.5926	716.3990	711.7958	716.4502	703.6014	716.0001
2	5.2526	696.3519	719.3682	714.7354	719.4217	706.4735	718.9741
3	5.2672	699.1164	722.3444	717.6817	722.4001	709.3517	721.9543
4	5.2817	701.8860	725.3277	720.6347	725.3855	712.2361	724.9407
5	5.2963	704.6607	728.3180	723.5944	728.3779	715.1267	727.9332
6	5.3108	707.4406	731.3153	726.5608	731.3772	718.0234	730.9318
7	5.3253	710.2255	734.3197	729.5340	734.3835	720.9263	733.9366
8	5.3399	713.0156	737.3313	732.5139	737.3968	723.8355	736.9475
9	5.3544	715.8108	740.3499	735.5006	740.4171	726.7508	739.9646
10	5.3690	718.6112	743.3755	738.4940	743.4443	729.6723	742.9879
11	5.3835	721.4166	746.4083	741.4942	746.4786	732.6000	746.0173
12	5.3980	724.2272	749.4483	744.5012	749.5199	735.5339	749.0529
13	5.4126	727.0429	752.4953	747.5149	752.5682	738.4741	752.0946
14	5.4271	729.8637	755.5495	750.5355	755.6235	741.4205	755.1425
15	5.4417	732.6897	758.6108	753.5628	758.6859	744.3730	758.1966
16	5.4562	735.5208	761.6793	756.5969	761.7553	747.3319	761.2568
17	5.4707	738.3570	764.7549	759.6379	764.8318	750.2969	764.3232
18	5.4853	741.1983	767.8377	762.6856	767.9153	753.2682	767.3957
19	5.4998	744.0447	770.9277	765.7402	771.0059	756.2457	770.4745
20	5.5144	746.8963	774.0248	768.8016	774.1036	759.2295	773.5593
21	5.5289	749.7530	777.1292	771.8698	777.2083	762.2195	776.6504
22	5.5435	752.6148	780.2407	774.9449	780.3202	765.2158	779.7476
23	5.5580	755.4817	783.3595	778.0269	783.4391	768.2183	782.8511
24	5.5725	758.3538	786.4855	781.1157	786.5652	771.2271	785.9606
25	5.5871	761.2310	789.6187	784.2113	789.6984	774.2421	789.0764
26	5.6016	764.1133	792.7592	787.3138	792.8387	777.2635	792.1983
27	5.6162	767.0007	795.9069	790.4233	795.9861	780.2911	795.3265
28	5.6307	769.8932	799.0619	793.5395	799.1407	783.3250	798.4608
29	5.6452	772.7909	802.2242	796.6627	802.3025	786.3652	801.6012
30	5.6598	775.6937	805.3937	799.7928	805.4714	789.4117	804.7479
31	5.6743	778.6016	808.5705	802.9298	808.6474	792.4645	807.9007
32	5.6889	781.5146	811.7546	806.0737	811.8307	795.5235	811.0598
33	5.7034	784.4328	814.9460	809.2246	815.0211	798.5889	814.2250
34	5.7179	787.3560	818.1448	812.3823	818.2187	801.6606	817.3964
35	5.7325	790.2844	821.3508	815.5470	821.4235	804.7386	820.5740
36	5.7470	793.2180	824.5642	818.7186	824.6355	807.8230	823.7578
37	5.7616	796.1566	827.7849	821.8972	827.8548	810.9136	826.9478
38	5.7761	799.1003	831.0130	825.0828	831.0812	814.0106	830.1440
39	5.7906	802.0492	834.2484	828.2753	834.3149	817.1139	833.3464
40	5.8052	805.0032	837.4912	831.4748	837.5559	820.2236	836.5549
41	5.8197	807.9623	840.7414	834.6812	840.8040	823.3396	839.7697
42	5.8343	810.9266	843.9989	837.8946	844.0595	826.4620	842.9907
43	5.8488	813.8959	847.2639	841.1151	847.3222	829.5907	846.2178
44	5.8634	816.8704	850.5362	844.3425	850.5922	832.7257	849.4512
45	5.8779	819.8500	853.8160	847.5770	853.8694	835.8672	852.6908
46	5.8924	822.8347	857.1032	850.8184	857.1540	839.0149	855.9366
47	5.9070	825.8246	860.3978	854.0669	860.4458	842.1691	859.1886
48	5.9215	828.8195	863.6999	857.3224	863.7449	845.3296	862.4468
49	5.9361	831.8196	867.0094	860.5849	867.0514	848.4966	865.7112
50	5.9506	834.8248	870.3263	863.8545	870.3652	851.6699	868.9818
Polynomial fit coefficients au ^2 + bu + c		a	17.1346	16.2586	16.8620	14.8417	14.6111
		b	24.3018	31.4853	27.3359	41.7445	51.2289
		c	118.9803	100.7827	110.6150	77.7237	46.7661
Target pace (m/s)		5.86042	849.8773	843.6925	849.9336	832.0940	848.7993

**Table 11:** Power values for two-pacer formations (2 and 12)

			Formation 2			Formation 12		
Interval	U	PR	Kip	PI	P2	Kip	PI	P2
1	5.2381	693.5926	707.0933	718.4411	718.7363	701.8815	715.5527	703.3460
2	5.2526	696.3519	709.9764	721.4128	721.7066	704.7221	718.4866	706.2112
3	5.2672	699.1164	712.8653	724.3908	724.6832	707.5689	721.4276	709.0820
4	5.2817	701.8860	715.7601	727.3752	727.6659	710.4218	724.3756	711.9587
5	5.2963	704.6607	718.6608	730.3658	730.6549	713.2810	727.3306	714.8411
6	5.3108	707.4406	721.5674	733.3628	733.6500	716.1464	730.2928	717.7293
7	5.3253	710.2255	724.4798	736.3660	736.6514	719.0180	733.2620	720.6233
8	5.3399	713.0156	727.3982	739.3756	739.6590	721.8958	736.2383	723.5230
9	5.3544	715.8108	730.3225	742.3915	742.6727	724.7798	739.2217	726.4285
10	5.3690	718.6112	733.2526	745.4138	745.6927	727.6701	742.2123	729.3398
11	5.3835	721.4166	736.1887	748.4423	748.7189	730.5667	745.2099	732.2569
12	5.3980	724.2272	739.1307	751.4772	751.7514	733.4695	748.2147	735.1797
13	5.4126	727.0429	742.0786	754.5184	754.7900	736.3785	751.2267	738.1083
14	5.4271	729.8637	745.0324	757.5660	757.8349	739.2938	754.2458	741.0427
15	5.4417	732.6897	747.9921	760.6199	760.8859	742.2154	757.2720	743.9829
16	5.4562	735.5208	750.9577	763.6801	763.9433	745.1432	760.3055	746.9289
17	5.4707	738.3570	753.9292	766.7467	767.0068	748.0773	763.3461	749.8807
18	5.4853	741.1983	756.9066	769.8196	770.0766	751.0177	766.3939	752.8382
19	5.4998	744.0447	759.8900	772.8988	773.1526	753.9645	769.4490	755.8015
20	5.5144	746.8963	762.8793	775.9845	776.2348	756.9175	772.5112	758.7707
21	5.5289	749.7530	765.8745	779.0765	779.3233	759.8768	775.5807	761.7456
22	5.5435	752.6148	768.8756	782.1748	782.4180	762.8424	778.6575	764.7263
23	5.5580	755.4817	771.8827	785.2795	785.5190	765.8143	781.7414	767.7128
24	5.5725	758.3538	774.8957	788.3906	788.6262	768.7926	784.8327	770.7051
25	5.5871	761.2310	777.9146	791.5080	791.7396	771.7772	787.9312	773.7032
26	5.6016	764.1133	780.9395	794.6318	794.8593	774.7681	791.0370	776.7071
27	5.6162	767.0007	783.9703	797.7620	797.9853	777.7653	794.1501	779.7169
28	5.6307	769.8932	787.0070	800.8986	801.1175	780.7690	797.2705	782.7324
29	5.6452	772.7909	790.0497	804.0415	804.2559	783.7789	800.3982	785.7537
30	5.6598	775.6937	793.0984	807.1909	807.4007	786.7952	803.5333	788.7808
31	5.6743	778.6016	796.1529	810.3466	810.5516	789.8179	806.6757	791.8138
32	5.6889	781.5146	799.2135	813.5087	813.7089	792.8470	809.8254	794.8525
33	5.7034	784.4328	802.2799	816.6772	816.8724	795.8824	812.9825	797.8971
34	5.7179	787.3560	805.3524	819.8521	820.0422	798.9243	816.1469	800.9475
35	5.7325	790.2844	808.4308	823.0334	823.2182	801.9725	819.3188	804.0037
36	5.7470	793.2180	811.5151	826.2211	826.4006	805.0271	822.4980	807.0657
37	5.7616	796.1566	814.6054	829.4152	829.5891	808.0881	825.6846	810.1335
38	5.7761	799.1003	817.7017	832.6157	832.7840	811.1555	828.8786	813.2071
39	5.7906	802.0492	820.8039	835.8226	835.9852	814.2293	832.0801	816.2866
40	5.8052	805.0032	823.9121	839.0360	839.1926	817.3096	835.2890	819.3719
41	5.8197	807.9623	827.0263	842.2557	842.4063	820.3963	838.5053	822.4630
42	5.8343	810.9266	830.1464	845.4819	845.6263	823.4894	841.7291	825.5600
43	5.8488	813.8959	833.2725	848.7145	848.8526	826.5890	844.9603	828.6628
44	5.8634	816.8704	836.4046	851.9536	852.0852	829.6950	848.1990	831.7714
45	5.8779	819.8500	839.5427	855.1990	855.3241	832.8074	851.4452	834.8858
46	5.8924	822.8347	842.6867	858.4509	858.5692	835.9263	854.6989	838.0061
47	5.9070	825.8246	845.8368	861.7093	861.8207	839.0517	857.9601	841.1322
48	5.9215	828.8195	848.9928	864.9740	865.0785	842.1835	861.2288	844.2641
49	5.9361	831.8196	852.1548	868.2452	868.3426	845.3218	864.5051	847.4019
50	5.9506	834.8248	855.3228	871.5229	871.6129	848.4666	867.7889	850.5455
Polynomial fit coefficients au ^2 + bu + c		a	14.0389	15.0722	14.7810	14.9886	17.2367	13.7204
		b	50.9589	46.2060	49.1776	38.0151	20.7817	53.0787
		c	54.9743	62.8680	55.5855	91.5092	133.7724	48.8618
Target pace (m/s)		5.8604	835.7733	851.3007	851.4336	829.0693	847.5471	831.1447

**Table 12:** Power values for three-pacer formations (3 and 6)

			Formation 3				Formation 6			
Interval	U	PR	Kip	PI	P2	P3	Kip	PI	P2	P3
1	5.2381	693.5926	706.2239	720.0656	720.3980	718.5714	703.1811	717.4722	717.8499	704.2581
2	5.2526	696.3519	709.0996	723.0318	723.3741	721.5427	706.0617	720.4596	720.8371	707.1313
3	5.2672	699.1164	711.9824	726.0058	726.3581	724.5217	708.9483	723.4536	723.8309	710.0108
4	5.2817	701.8860	714.8722	728.9877	729.3500	727.5083	711.8410	726.4543	726.8312	712.8966
5	5.2963	704.6607	717.7691	731.9774	732.3498	730.5027	714.7399	729.4615	729.8381	715.7886
6	5.3108	707.4406	720.6732	734.9750	735.3577	733.5047	717.6448	732.4753	732.8516	718.6869
7	5.3253	710.2255	723.5843	737.9805	738.3734	736.5145	720.5558	735.4957	735.8716	721.5916
8	5.3399	713.0156	726.5025	740.9938	741.3972	739.5319	723.4729	738.5228	738.8982	724.5024
9	5.3544	715.8108	729.4279	744.0151	744.4290	742.5572	726.3962	741.5565	741.9313	727.4196
10	5.3690	718.6112	732.3604	747.0443	747.4688	745.5901	729.3255	744.5968	744.9710	730.3431
11	5.3835	721.4166	735.3001	750.0815	750.5166	748.6309	732.2610	747.6437	748.0173	733.2730
12	5.3980	724.2272	738.2469	753.1266	753.5725	751.6794	735.2025	750.6973	751.0702	736.2091
13	5.4126	727.0429	741.2010	756.1797	756.6365	754.7357	738.1502	753.7575	754.1297	739.1515
14	5.4271	729.8637	744.1622	759.2408	759.7085	757.7998	741.1041	756.8244	757.1958	742.1003
15	5.4417	732.6897	747.1306	762.3099	762.7886	760.8718	744.0640	759.8979	760.2685	745.0554
16	5.4562	735.5208	750.1062	765.3871	765.8769	763.9516	747.0301	762.9781	763.3478	748.0169
17	5.4707	738.3570	753.0891	768.4723	768.9733	767.0393	750.0023	766.0650	766.4336	750.9847
18	5.4853	741.1983	756.0792	771.5655	772.0779	770.1348	752.9807	769.1585	769.5261	753.9588
19	5.4998	744.0447	759.0765	774.6668	775.1906	773.2382	755.9652	772.2587	772.6253	756.9393
20	5.5144	746.8963	762.0811	777.7762	778.3115	776.3495	758.9558	775.3655	775.7310	759.9261
21	5.5289	749.7530	765.0930	780.8938	781.4406	779.4687	761.9526	778.4791	778.8434	762.9193
22	5.5435	752.6148	768.1122	784.0194	784.5779	782.5958	764.9556	781.5993	781.9624	765.9189
23	5.5580	755.4817	771.1387	787.1532	787.7234	785.7309	767.9647	784.7262	785.0880	768.9249
24	5.5725	758.3538	774.1725	790.2952	790.8772	788.8740	770.9800	787.8598	788.2203	771.9372
25	5.5871	761.2310	777.2136	793.4453	794.0393	792.0250	774.0014	791.0001	791.3592	774.9560
26	5.6016	764.1133	780.2620	796.6036	797.2097	795.1840	777.0290	794.1472	794.5047	777.9811
27	5.6162	767.0007	783.3178	799.7701	800.3883	798.3510	780.0628	797.3009	797.6569	781.0126
28	5.6307	769.8932	786.3810	802.9449	803.5753	801.5260	783.1027	800.4613	800.8158	784.0505
29	5.6452	772.7909	789.4515	806.1278	806.7706	804.7090	786.1488	803.6285	803.9813	787.0949
30	5.6598	775.6937	792.5295	809.3191	809.9742	807.9000	789.2011	806.8023	807.1535	790.1456
31	5.6743	778.6016	795.6148	812.5186	813.1862	811.0992	792.2596	809.9829	810.3323	793.2028
32	5.6889	781.5146	798.7075	815.7264	816.4066	814.3064	795.3243	813.1703	813.5179	796.2664
33	5.7034	784.4328	801.8077	818.9425	819.6354	817.5216	798.3952	816.3643	816.7101	799.3364
34	5.7179	787.3560	804.9153	822.1669	822.8726	820.7450	801.4723	819.5652	819.9089	802.4129
35	5.7325	790.2844	808.0303	825.3996	826.1182	823.9765	804.5555	822.7727	823.1145	805.4958
36	5.7470	793.2180	811.1528	828.6407	829.3723	827.2161	807.6450	825.9870	826.3268	808.5851
37	5.7616	796.1566	814.2828	831.8902	832.6349	830.4639	810.7407	829.2081	829.5457	811.6809
38	5.7761	799.1003	817.4202	835.1480	835.9059	833.7198	813.8426	832.4359	832.7714	814.7832
39	5.7906	802.0492	820.5652	838.4143	839.1854	836.9839	816.9507	835.6705	836.0037	817.8919
40	5.8052	805.0032	823.7176	841.6889	842.4734	840.2561	820.0650	838.9119	839.2428	821.0071
41	5.8197	807.9623	826.8776	844.9720	845.7700	843.5366	823.1855	842.1600	842.4886	824.1288
42	5.8343	810.9266	830.0451	848.2636	849.0751	846.8253	826.3123	845.4149	845.7411	827.2569
43	5.8488	813.8959	833.2202	851.5636	852.3888	850.1222	829.4453	848.6766	849.0003	830.3915
44	5.8634	816.8704	836.4028	854.8720	855.7110	853.4273	832.5845	851.9451	852.2662	833.5327
45	5.8779	819.8500	839.5929	858.1890	859.0419	856.7407	835.7299	855.2204	855.5389	836.6803
46	5.8924	822.8347	842.7907	861.5145	862.3813	860.0624	838.8816	858.5024	858.8183	839.8344
47	5.9070	825.8246	845.9961	864.8485	865.7294	863.3924	842.0396	861.7913	862.1044	842.9951
48	5.9215	828.8195	849.2090	868.1911	869.0861	866.7306	845.2037	865.0870	865.3973	846.1622
49	5.9361	831.8196	852.4296	871.5422	872.4515	870.0772	848.3742	868.3895	868.6969	849.3359
50	5.9506	834.8248	855.6578	874.9019	875.8255	873.4321	851.5509	871.6988	872.0033	852.5161
Polynomial fit coefficients au ^2 + bu + c		a	17.365648	19.3857	19.606242	18.896746	14.589083	15.858832	15.723154	15.123398
		b	15.401443	0.3718697	-1.266629	5.8815245	44.996361	39.006687	40.422894	38.856084
		c	149.08848	186.2347	189.09882	169.29449	67.199147	78.026911	74.708725	85.780988
Target pace (m/s)		5.86042	835.76231	854.20662	855.04286	852.7624	831.95183	851.28648	851.60807	832.89986

## Appendix 3

In this section, the evolution of drafting and pacing strategies is presented. Originally, this was part of the main paper; it has been transferred for the sake of briefness. The main body of the manuscript now features only the necessary information.

It is worth mentioning that the power calculations used in this section were performed using the model by Cavagna and Kaneko [39], also employed in previous research [25, 28]. The final results were calculated using a more realistic model [44].

### Calculation of Power Values

One exemplary application of the mechanical power model is as follows: the necessary speed to cover a marathon distance (42.195 km) in exactly two hours is 21.0975 $$km/h$$ (5.86 $$m/s).$$ Substituting that speed in Eq. (2) yields the specific running power, $${P}_{R}$$ in $$cal/s,$$ as a function of the mass of the runner.

14 $${P}_{R}=225.108 m$$

We use a value of 52 kg for Eliud Kipchoge [53]. Substituting that in the previous equation yields the total estimated running power of ${P}_{R}=11705.7 cal/s (816.820 W)$. As stated previously, additional energy is used to overcome drag. This portion, called Aerodynamic power, ${P}_{A}$, is defined as

15 $${P}_{A}={F}_{D}\cdot u=\frac{1}{2}\rho {u}^{3}{C}_{D}A$$

where ${F}_{d}$ is the drag force, $\rho$ is the air density, ${C}_{d}$ is the drag coefficient and $A$ is the projected frontal area of the runner. The running velocity, $u$, changes between different running events (and even during different phases within the same event). The area, $A$ is the projected area of the runner (“Appendix 1”).

The drag force, ${F}_{d},$ and coefficient, ${C}_{d}$, are calculated from the CFD simulations.

The experiments for energy expenditure were performed on treadmills, where the air was stagnant and no form drag was present; in order to account for the air flow component of energy expenditure, the total running power, ${P}_{tot}$, must also include the aerodynamic power:

16 $${P}_{tot}={P}_{R}+{P}_{A}$$

In order to facilitate our analysis, we shall simplify the equation for ${P}_{R}$ to ensure that the units are consistent. The mass has been substituted, and all the velocity values are in $m/s$. Power values are calculated in $W$.

17 $${P}_{R}=34.158+61.745u+12.388{u}^{1.993}$$

The aerodynamic power, ${P}_{A}$, is still calculated as ${F}_{d}\cdot u$, with ${F}_{D}$ in $N$ and $u$ in $m/s$

Here, it must be considered that the total power produced by the body during a run is bigger than ${P}_{tot}$, due to metabolic efficiency, $\epsilon$, which varies according to running speed. The metabolic power is defined as

18 $${P}_{meta}=\frac{{P}_{tot}}{\epsilon }$$

This phenomenon has been studied extensively in the past, though the efficiency and mechanical models are inherently difficult to define. In the present work, we opted to use a fixed running efficiency, consistent with the work done by Schickhofer and Hanson [28], who adopted a value of 0.64.

### Energy Considerations

In the previous section, we made qualitative considerations about the benefits of drafting, paving the way for potential time savings associated with the use of more aerodynamically effective formations. We shall attempt to break the sub 2-h barrier for official marathon events, the 2018 and 2022 editions of the Berlin marathon.

In order to calculate the time improvements, power values must be calculated. The evolution of our pacing strategy shown in the next section, highlights the necessity of considering the energy expenditure of not only the main runner (Kipchoge) but also of his pacers in order to achieve better results. Therefore, the drag calculations must be expanded for every runner in the formation.

Second degree polynomial fits were calculated for all runners and formations with great accuracy. These functions allow us to calculate the aerodynamic power for any velocity within the limits ${u}_{a}$ and ${u}_{d}$. This interval was divided into 50 velocity values, at which the total running power for each runner was calculated. The results are present in “Appendix 2” (Tables 10, 11 and 12 are also present in the supplementary materials). From these values, and for each formation, a second order polynomial fit was performed with great accuracy, allowing for a fast calculation of the running power for any velocity.

### Evolution of the Pacing Strategies

In this section, the lengthy evolution of our pacing strategy is discussed in detail. In case the reader is interested only in the sub 2 h pacing strategy, we recommend skipping to “Section Conservation of Energy and Performance Projections”. However, each of the failed attempts provided the authors with valuable insight which led to further development.

For iterations 1 and 2, the first step is the division of each race into the appropriate time periods when different formations were used; the results for the 2018 Berlin marathon are shown in Tables 1; Table 2 shows the pertinent data for the 2022 Berlin marathon. The earliest iterations were evaluated for the 2018 Berlin marathon, unless otherwise noted.

The first two iterations only make energy considerations about the main runner, disregarding possible energy savings by the pacers.

The second step is to identify the portions of the race where improvements were possible. In this case, Formation 6 was qualitatively determined to be more effective than formation 3, with the same happening with Formations 4 and 1 (at this point, Formation 12 hadn’t been investigated yet, therefore we neglected possible improvements in the two-pacer phase).

It is evident that a more effective formation would result in time savings, as long as the number of pacers is the same for both. There are two ways to proceed with the analysis at this point. The first iteration, although intuitive, has a major flaw which increases its complexity; however, it will be shown below, to help illustrate our preference for the second method.

### Iteration 1—Change the Formations Within Each Phase of the Race, Keeping the Distance Constant

The third step is to calculate the total mechanical power output for the desired phase and compare it with the output for the improved formation. The relevant equations are repeated below:

19 $${P}_{R}=34.158+61.745u+12.388{u}^{1.993}$$

20 $${P}_{A}={F}_{d}\cdot u$$

21 $${P}_{tot}={P}_{R}+{P}_{A}$$

Substituting the appropriate values for the period of time when Formation 1 yields the power values for that portion of the race. The velocity, $u$, is calculated from Table 1; the expression of the drag force as a function of the velocity is the second order fit, presented in Table 10.

22 $${\left.{P}_{R}\right|}_{Berlin 2018}^{formation 1}=792.779 W$$

23 $${\left.{P}_{A}\right|}_{Berlin 2018}^{formation 1}=25.723 W$$

24 $${\left.{P}_{tot}\right|}_{Berlin 2018}^{formation 1}= 818.532 W$$

The fourth step is to apply the conservation of total mechanical power output for that phase of the race. In this specific case, improved aerodynamic performance would be calculated by the use of Formation 4 instead of 1. The new velocity at which Kipchoge would be able to run at is unknown at this point and is denoted $v$. The power values must be expressed in terms of that variable:

25 $${\left.{P}_{R}\right|}_{Berlin 2018}^{formation 4}=34.158+61.745v+12.388{v}^{1.993}$$

26 $${\left.{P}_{A}\right|}_{Berlin 2018}^{formation 4}={F}_{d}\left(v\right)\cdot v$$

We do not have a direct expression for ${P}_{A}$, since $v$ is not known a priori. However, we do have the expression for ${F}_{d}$ as a function of $v$ (Table 9):

27 $${F}_{d,4}=0.218{v}^{2}-1.473v+3.613$$

The conservation of total power output yields the following expression:

28 $$34.158+61.745v+12.388{v}^{1.993}+\left(0.218{v}^{2}-1.473v+3.613\right)\cdot v=818.532$$

This equation is then solved numerically, resulting in $v=5.803 m/s.$

The final step is to calculate the new time to cover the distance elapsed during that phase of the race and compare that to the actual time elapsed. In this case, the same savings associated with the use of Formation 4 instead of Formation 1 during the 9.99 km phase of the race are $24.88$s. That analysis can then be expanded to all the phases of the race, resulting in a calculation of total time savings.

In this method, the implicit assumption is that the pacer would not have quit up to the end of that phase of the race, despite running at a faster pace. This is obviously false, since his running power will be higher (higher running velocity) and the aerodynamic power will also be higher (again, due to a higher drag force caused by a running velocity). Therefore, by using this method, we would be expecting all pacers to deliver more total power than they did, which is unrealistic. Another alternative is needed, one that preserves the total power output of both Kipchoge and all pacers. The procedure is detailed below.

### Iteration 2—Change the Formations Within Each Phase of the Race, Keeping the Velocity Constant

In the second alternative, the idea is to calculate the total power output of Kipchoge (for the entire race). We assume that the first three phases of the run (Formations 3, 2 and 1, respectively) would be run at the same pace as in the actual race, despite the different formations. This way, the pacers would have the same (or even lower) instantaneous and total power outputs during the race. For this analysis to be accurate, the drag experienced by the pacers in the different formations has to be similar, which lead us to modify the CFD simulations to also calculate the drag experienced by the pacers.

For instance, the drag force experienced by the sole pacer in Formation 4 is actually lower than in Formation 1 (Table 10). The lateral offset in Kipchoge’s position only causes a slight variation in the drag experienced by the pacer. The plots are not shown here (please refer to Table 11), but a similar effect happens between Formations 3, 5 and 6, with all pacers facing lower amounts of drag.

The use of this approach also makes sense from a strategic standpoint; after all, the presence of the pacers primarily meets the purpose of keeping Kipchoge’s pace constant (and not to reduce the drag experienced by him). In iteration 1, we assumed that each portion of the race could be run faster by preserving instantaneous mechanical power and using a more effective formation.

In iteration 2, rather than modifying the velocity of each phase of the race, we calculate the energy savings in each portion by keeping the velocity constant and using the more aerodynamically effective formation—while assuming the pacers have enough power to cover. This hypothesis holds true because the drag values of the pacers are always lower or very close in the equivalent formations. Also, the pacers would exit at the same time they did in the original race.

The energy savings can then be used to run the final portion of the race (when no pacers are present) at a higher speed. The procedure is outlined with a continuation of the global steps described previously.

The third step is to calculate Kipchoge’s power output for the entire race. The results are presented in Table 13 and were calculated using the equations for running power and aerodynamic power (“Section Methods”). The drag force is calculated from the second order fit (Table 9). At this point, data for Formation 2 is omitted since this portion does not change between the actual and projected races, and no energy savings exist (again, Formation 12 hadn’t been considered yet).

**Table 13:** Power values for Eliud Kipchoge during the 2018 Berlin marathon

Time elapsed	Distance run (km)	$u(m/s)$	Formation	${F}_{d} (N)$	${P}_{tot}(W)$	$E({10}^{6}J)$
00:41:17	14.23	5.744	3	$3.182$	$811.065$	$2.009$
00:04:22	1.46	5.572	2	–	–	–
00:28:44	9.99	5.794	1	$4.582$	$829.421$	$1.429$
00:47:16	16.515	5.823	Solo	$5.641$	$841.554$	$2.386$
02:01:39	42.195	5.780	–	–	–	$5.825$

The fourth step is a repetition of the third step with the modified formations. In that case, Formation 3 was substituted by Formation 6 and Formation 1 was substituted by Formation 4. Results are shown in Table 14. The altered formations, drag, power and energy values are highlighted.

**Table 14:** Predicted power values for Eliud Kipchoge during the 2018 Berlin marathon

Time elapsed	Distance run (km)	$u(m/s)$	Formation	${F}_{d} (N)$	${P}_{tot}(W)$	$E({10}^{6}J)$
00:41:17	14.23	5.744	6	$2.3437$	$805.885$	$1.996$
00:04:22	1.46	5.572	2	–	–	–
00:28:44	9.99	5.794	4	$2.3941$	$816.453$	$1.408$
$t$	16.515	$v$	Solo	${F}_{d}(v)$	${P}_{tot}(v)$	$E(v)$
–	42.195	–	–	–	–	$5.825$

The elapsed time and velocity for the solo runner phase are the variables that must be determined. The drag value, as well as total power and energy values, are functions of the unknown velocity. However, the total energy expenditure during the run is known, from which $E(v)$ is determined:

29 $$E\left(v\right)=5.825-1.966-1.408=2.421\cdot {10}^{6} J$$

The average power output, ${P}_{tot}$, is calculated by dividing the total energy for the time elapsed. The time elapsed is a function of distance and velocity, $t=d/v$. The distance $d$ is $16515 m$.

30 $${P}_{tot}\left(v\right)=\frac{E(v)}{t}=\frac{E\left(v\right)\cdot v}{d}$$

Then, we expand ${P}_{tot}$ in its two components, ${P}_{R}$ and ${P}_{A}$, using the equations from “Appendix 3”:

31 $${P}_{tot}\left(v\right)={P}_{R}\left(v\right)+{P}_{A}\left(v\right)$$

32 $$\frac{E\left(v\right)\cdot v}{d}=34.158+61.745v+12.388{v}^{1.993}+{F}_{d}\left(v\right)\cdot v$$

The last step is to substitute the appropriate expression for ${F}_{d}(v)$, according to the appropriate formation. In this case (Solo runner), the coefficients are present in Table 9. The values for $E(v)$ and $d$ can also be substituted:

33 $$\frac{2.408\cdot {10}^{6}\cdot v}{16515}=34.15+61.74v+12.38{v}^{1.993}+\left(0.493{v}^{2}-3.256v+7.878\right)\cdot v$$

Finally, this equation is solved numerically. The solution is $v=5.9764 m/s$, meaning that the final $16.515 km$ would have been covered in 2763 s (46:03). Compared to the original elapsed time of 47:16 for that phase, the new pacing strategy resulted in 72 s being saved, which would account for an overall time of 2:00:25. This is a great improvement over the initial time of 2:01:39, although not quite the desired result. One thing to notice is that Kipchoge would have to accelerate a lot in the final portion of the race, which might not be realistically possible.

This analysis was also performed for the 2022 Berlin marathon. The running splits, drag forces and power values are shown in Table 15.

**Table 15:** Running splits and power values for Eliud Kipchoge during the 2022 Berlin marathon

Time elapsed	Distance run (km)	$u(m/s)$	Formation	${F}_{d} (N)$	${P}_{tot}(W)$	$E({10}^{6}J)$
01:04:08	22.59	5.870	3	$3.359$	$838.080$	$3.224$
00:06:00	2.07	5.750	1	$4.493$	$819.659$	$0.295$
00:51:01	17.535	5.728	Solo	$5.409$	$820.479$	$2.511$
02:01:09	42.195	5.804	–	–	–	$6.0314$

The formation changes are highlighted in Table 16.

**Table 16:** Predicted power values for Eliud Kipchoge during the 2022 Berlin marathon

Time elapsed	Distance run (km)	$u(m/s)$	Formation	${F}_{d} (N)$	${P}_{tot}(W)$	$E({10}^{6}J)$
01:04:08	22.59	5.8705	6	$2.442$	$832.695$	$3.204$
00:06:00	2.07	5.750	4	$2.346$	$807.315$	$2.906$
$t$	17.535	$v$	Solo	${F}_{d}(v)$	${P}_{tot}(v)$	$E(v)$
–	42.195	–	–	–	–	$6.031$

Now, the procedure performed for the 2018 Berlin marathon is repeated. Since the sequence has already been demonstrated, the calculations are omitted. The resulting velocity for the final phase is $v=5.834 m/s$, which would have resulted in a savings of $55$ seconds (for a 2:00:14 finish).

Overall, the predicted results for the 2018 Berlin marathon (2:00:25) and for the 2022 Berlin marathon (2:00:14) at this point are remarkable (both would have been substantial improvements to the world records by Kipchoge at the time of the races). However, the authors were struck with the intuition that something else could be done to further reduce the times, possibly reaching the sub 2 h mark.

### Developments After Iterations 1 and 2

In the previous attempts, an effort was made to maintain either the distance or the velocity of most parts of the races constant. Initially, for the attempt with fixed distances, the effects of the modified formations were ignored totally. Then, an effort was made to show that the pacers could keep running at the same pace as in the original race in the modified formations. This allowed us to predict 2:00:25 and 2:00:14 finishes for the 2018 and 2022 editions of the Berlin marathon, respectively. Still, the presence of the pacers didn’t receive our full attention (Fig. 18).

One thing about Formation 6 caught our attention: unlike in Formation 3 (where all pacers are directly facing the wind), Formation 6 affords the pacer running left of Kipchoge (P3) the same drag benefits experienced by the main runner (Fig. 19). This raised the possibility of that pacer staying in the race for longer (since he also develops a lower running power).

If a combination of formations to achieve a sub 2 h result is indeed possible, we were convinced that this would require a team effort, with all pacers playing a key role. It was necessary, then, to further modify our models to include the presence of all pacers in the most accurate way possible.

This was achieved through calculations of mechanical power for all pacers in the relevant formations, in addition to Kipchoge. The results are presented in Tables 10, 11 and 12. For a group effort, the total energy expenditure needs to be considered, and not just the one of the main runner.

Also, a comment has to be made about pacing: in Kipchoge’s attempts, the pacers were used more to help him stay at the desired pace than to reduce drag (which is clear by the sub-optimal formations employed by the team in both attempts). However, in both races, Kipchoge’s velocity fluctuated throughout the race, with some portions being run faster than others.

Every time a runner accelerates, further energy is spent. For best results, the pace should be kept as close as possible to a constant desired value. This idea was successfully used in the INEOS 2019 challenge, where the pace stayed virtually constant for the duration of the race. At this point, it was clear that a successful pacing/drafting strategy for the sub 2 h marathon would be centered around a target pace.

One next modification was the inclusion of Formation 12, consisting of a three-man line with the main runner in the middle (Fig. 20).This formation was featured in [28] and caught our attention for the same reason as Formation 6: the pacer behind Kipchoge (P2) is also afforded a drag benefit.

We have already established that the best Formation for 3 pacers is Formation 6, and that the best formation for one pacer is Formation 4; This is confirmed by the results of total power output by the runners.

However, quite surprisingly, upon comparison of Formations 6 and 12, it turns out that Formation 12 can have a competitive advantage over all the other formations when two pacers are available. Upon careful evaluation of Tables 11 and 12, it is clear that both pacers in Formation 12 are also required to develop less power than any of the three pacers in Formation 6 at the same speed.

This leads to a rather counterintuitive consequence: when three pacers are available, it is better to use only two of them from an energy standpoint. The cornerstone of our sub 2 h pacing strategy is to begin the race in this formation. Out of the three available pacers, the two with the greater endurance must be chosen. Kipchoge and his trainers are reasonably expected to know this information, since he and the pacers train together before the race.

One further detail about Formation 12 is worth noticing: pacer P1, who runs in front, has a higher mechanical power output. Two pacers are available (one of which will leave the race first). The question of who should be P1 is an interesting one. We can either place the stronger pacer in that position, thus keeping P2 in the race for longer, or protect the stronger pace in the P2 position, theoretically meaning that at least one pacer stays in the race for longer. Both options will be investigated.

The lessons learned in the previous attempts, along with the recently made observations, result in a new strategy:

- Start the race in Formation 12 and maintain that formation for as long as two pacers stay in the race; the running velocity should be $u=5.86 m/s$, enough to cover the marathon distance in exactly two hours.
- As soon as the first pacer leaves the race, switch to Formation 4 and remain in it for as long as the (now solo) pacer remains in the race; continue running at the same pace;
- When the last pacer drops out, finish the race alone at the same speed (or faster, depending on the energy available), hopefully finishing in under two hours.

The analysis starts with the calculation of mechanical energy spent by each runner in each phase of the race, as shown in Table

**Table 17:** Energy spent by each runner during the 2018 Berlin marathon $({10}^{6}J)$

Time elapsed	Formation	Kipchoge	Boit	Kitwara	Kipkemboi
00:41:17	3	2.008	2.050	2.050	2.050
00:04:22	2	0.203	0.206	0.206	–
00:28:44	1	1.429	1.439	–	–
00:47:16	Solo	2.386	–	–	–
02:01:39	–	6.027	3.697	2.257	2.050

^17^. To avoid confusion with the numbers assigned to the pacers, the three pacers are identified by their names: Sammy Kitwara, Bernard Kipkemboi and Josphat Boit.

We shall investigate two possibilities, alternating the position of the pacer who runs in front in Formation 1. For both cases, the running velocity is constant ($$u=5.860 m/s)$$ until no pacers remain. For both cases, only the two strongest pacers will be included (Boit and Kitwara).

### Iteration 3—Starting the Race in Formation 12, with the Best Pacer (Boit) Running in the P1 Position and the Second-Best Pacer (Kitwara) in the P2 Position

The time spent in Formation 12 is limited by how long the weaker pacer (Kitwara) can sustain his P2 position at $5.860 m/s$. According to Table 11, his total running power for that position and pace is $831.144 J$.

The time spent in the first phase is, then:

34 $${t}_{1}=\frac{2257367}{831.144}=2715.973 s\approx 45:16$$

After that period, only Boit remains, and the two runners should switch to Formation 1. The information in Table 11 allows us to determine the energy spent by Kipchoge and Boit during the first phase:

35 $${E}_{1}^{Kipchoge}=829.069\cdot {t}_{1}=2251729.833 J$$

36 $${E}_{1}^{Boit}=847.547\cdot {t}_{1}=2301915.039 J$$

The time spent in the second phase (Formation 1) is determined by how much energy remains for Boit:

37 $${E}_{2}^{Boit}=\left(3.697-2.301\right)\cdot {10}^{6}=1.395\cdot {10}^{6} J$$

According to Table 10, Boit’s total running power for that position and pace is $848.7993 J$. The time spent in the second phase is, then:

38 $${t}_{2}=\frac{1.395\cdot {10}^{6}}{848.799}=1643.932 s\approx 27:24$$

The energy spent by Kipchoge during phase 2 is:

39 $$E_{2}^{{Kipchoge}} = 832.094 \cdot t_{2} = 1367905.953J$$

For the third phase, only Kipchoge remains. The remaining energy for that phase is:

40 $${E}_{3}^{Kipchoge}=\left(6.027-2.251-1.367\right)\cdot {10}^{6}=2.407\cdot {10}^{6} J$$

Before the calculation of the velocity for the third and final phase is possible, the cumulative distance and time up to that point must be calculated:

41 $${t}_{1+2}=2715.973+1643.932=4359.905 s\approx 1:12:39s$$

42 $${d}_{1+2}=u\cdot {t}_{1+2}=5.86\cdot 4359.905=25550.874 m$$

Which leads us to the remaining distance:

43 $${d}_{3}=42195-25550.874=16644.126 m$$

Assuming a velocity $v$ for the final phase, the time of the third phase is ${t}_{3}={d}_{3}/v$. Just like in the previous analysis, the running power is a function of the velocity. The energy spent by Kipchoge in the final phase is:

44 $${E}_{3}^{Kipchoge}={P}_{tot,Formation 0}\left(v\right)\cdot {t}_{3}={P}_{tot,Formation 0}\left(v\right)\frac{{d}_{3}}{v}$$

The solution for $v$ can be found either via numerical methods in MATLAB or through the use of a goal seek function in Excel. A script is provided in the supplementary materials. The values are found to be:

45 $$v=5.833\frac{m}{s}$$

46 $${t}_{3}=2853.238 s$$

Which leads to a total time of:

47 $${t}_{total}=7213.143s\approx 2:00:13$$

Which is a full 12 s faster than the previous best prediction, although not below the desired mark yet. Our next try is to switch the position of the pacers in Formation 12.

### Iteration 4—Starting the Race in Formation 12, with the Best Pacer (Boit) Running in the P2 Position and the Second Best Pacer (Kitwara) in the P1 position

The previous procedure is repeated with a slight tweak. The time spent in Formation 12 is limited by how long the weaker pacer (Kitwara) can sustain his P1 position at $5.8604 m/s$. According to Table 11, his total running power for that position and pace is $847.5471 J$.

The time spent in the first phase is, then:

48 $${t}_{1}=\frac{2257367}{847.547}=2663.411 s\approx 44:24$$

After that period, only Boit remains, and the two runners should switch to Formation 1. The information in Table 11 allows us to determine the energy spent by Kipchoge and Boit during the first phase:

49 $${E}_{1}^{Kipchoge}=829.069\cdot {t}_{1}=2208152.293 J$$

50 $${E}_{1}^{Boit}=831.144\cdot {t}_{1}=2213679.936 J$$

The time spent in the second phase (Formation 1) is determined by how much energy remains for Boit:

51 $${E}_{2}^{Boit}=\left(3.697-2.213\right)\cdot {10}^{6}=1.483\cdot {10}^{6} J$$

According to Table 10, Boit’s total running power for that position and pace is $848.7993 J$. The time spent in the second phase is, then:

52 $${t}_{2}=\frac{1.483\cdot {10}^{6}}{848.7993}=1747.886 s\approx 29:08$$

The energy spent by Kipchoge during phase 2 is:

53 $${E}_{2}^{Kipchoge}=832.094\cdot {t}_{2}=1454405.453 J$$

For the third phase, only Kipchoge remains. The remaining energy for that phase is:

54 $${E}_{3}^{Kipchoge}=\left(6.027-2.208-1.454\right)\cdot {10}^{6}=2.364\cdot {10}^{6}$$

Before the calculation of the velocity for the third and final phase can be made, the cumulative distance up to that point must be calculated:

55 $${d}_{1+2}=u\cdot {t}_{1+2}=5.860\cdot 4411.297=25852.053 m$$

Which leads us to the remaining distance:

56 $${d}_{3}=42195-25852.053=16342.947 m$$

Assuming a velocity $v$ for the final phase, the time of the third phase is ${t}_{3}={d}_{3}/v$. Just like in the previous analysis, the running power is a function of the velocity. The energy spent by Kipchoge in the final phase is:

57 $${E}_{3}^{Kipchoge}={P}_{tot,Formation 0}\left(v\right)\cdot {t}_{3}={P}_{tot,Formation 0}\left(v\right)\frac{{d}_{3}}{v}$$

The solution for $v$ can be found either via numerical methods in MATLAB or through the use of a goal seek function in Excel. The values are found to be:

58 $$v=5.836\frac{m}{s}$$

59 $${t}_{3}=2800.226 s$$

Which leads to a total time of:

60 $${t}_{total}=7211.523\approx 2:00:11$$

Which is a slight improvement over the previous case. At this point, one final observation was made: in both cases 1 and 2, Kipchoge would run at the desired 2 h pace (5.86042 m/s) whenever he was protected by at least one pacer, running the remainder of the race at the fastest possible pace given his remaining energy. However, when running alone, not only is the drag experienced much higher, but also the total running power. Since the running power grows as a function of ${u}^{2}$, it might actually be better to run the final portion of the race slower. The answer to this question is investigated in our final attempt to improve our strategy, iteration 5.

### Iteration 5—Refinement of Iteration 4, with Protected Portion of the Race Being Run at a Faster Speed

For the last iteration, we shall test a range of velocities for the protected phase (when at least one pacer is present), calculating the velocity for the unprotected phase (when Kipchoge runs alone) and the total race time. The complete procedure is presented in the next section.

### Sub 2-h Pacing Strategy for the 2022 Berlin Marathon

In this section, the culmination of the development of our drafting/pacing strategy is presented. We highlight that every choice made in the final strategy is the consequence of a lesson learned from a failed iteration. Also, this strategy was attempted for the 2018 Berlin marathon, resulting in a possible 2:00:10 finish. The guidelines are a set of rather counterintuitive (and yet, very simple) steps.

### Guidelines for a Sub 2 h Strategy

- Run the protected portion of the race (i.e., when at least one pacer is available) at a velocity ${v}_{i}$, faster than the unprotected portion of the race (when running alone at velocity ${v}_{f}$). Since the running power is roughly a function of ${v}^{2}$, it is preferable to run faster when the drag is lower (i.e., when running in any formation).
- Start the race in Formation 12 (a two-pacer formation), even if 3 pacers are available. The third pacer does not participate in the race at all. The two best pacers must be previously known by the running team. The pacer running in front of the formation (P1, Fig. 17, “Appendix 2”) has the highest power output among the three runners. The evolution of our strategy indicated that it is better that the strongest pacer runs in the protected position P2, so that he can stay in the race for longer.
- When the first pacer drops out, switch immediately to Formation 4. Maintain speed ${v}_{1}$ for as long as the remaining pacer continues in the race. As soon as the second pacer drops, continue the race alone at a velocity ${v}_{f}$, lower than ${v}_{i}.$

Once again, each of these decisions is based on the evolution of our drafting strategy. Now, we shall present all the relevant data and the procedure for calculation of the improved time.

### Application to the 2022 Berlin Marathon

The procedure will be divided into three major steps for clarification.

#### Step 1: evaluation of formations used during each running phase

The first step is to evaluate the race broadcast and determine for how long each formation was used. The results are presented in Table 2.

A very important observation should be made about these numbers. Upon careful evaluation of the race broadcast [33], it is clear that the first 22.59 km were covered in Formation 3, after which the first pacer dropped. Unfortunately, during the next 6 min the broadcast did not show Kipchoge enough for us to determine exactly when the second pacer dropped. It is known, however, that only one pacer remained after that period.

In order to proceed with the analysis, we made the conservative assumption that these 6 min were spent in Formation 1. This analysis is indeed conservative because, at the same speed, both Kipchoge and his pacer have a lower total power output in any of the 2-pacer formations when compared to Formation 1. Data for power output values is present in Table 10–12.

#### Step 2: calculation of energy output for each runner during the actual race

The energy values spent in each phase by each runner are calculated using the running and aerodynamic power functions (“Section Energetics of running”). The results are shown in Table

**Table 18:** Energy spent by each runner during the 2022 Berlin marathon $({10}^{6}\text{J})$

Time elapsed	Formation	Kipchoge	Kiplimo	Koech	Kipkemboi
01:04:08	3	3.224	3.295	3.295	3.295
00:06:00	1	0.294	0.297	–	–
00:51:01	Solo	2.511	–	–	–
02:01:09	–	6.031	3.592	3.295	3.295

^18^. The pacers are identified by their names: Isiah Koech, Bernard Kipkemboi and Jacob Kiplimo. Koech and Kipkemboi are assumed to have dropped out of the race together, according to our conservative approach. In our simulated attempt to break the 2 h barrier, the two pacers chosen are Kiplimo and one among Koech and Kipkemboi; we chose to move forward with Koech, without loss of generality.

#### Step 3: MATLAB implementation

Since neither ${v}_{i}$ or ${v}_{f}$ are known a priori, the conservation of energy for each runner must be implemented using variables. The script is provided in the supplementary materials, and each relevant portion is also explained below.

The race is split into three phases: phase 1, when Formation 12 employed, during ${t}_{1}$ seconds; phase 2, when Formation 4 is employed, during ${t}_{2}$ seconds; and, finally, phase 3, when Kipchoge runs alone.

Phase 1: race starts at velocity ${v}_{i}$ and Formation 12, with Koech as the lead pacer, Kipchoge in the middle and Kiplimo as the trailing pacer. The total power outputs for each of these running positions have been calculated as functions of running velocity (Table 11):

61 $${P}_{Koech,1}=17.236\cdot {v}_{i}^{2}+20.781\cdot {v}_{i}+133.772$$

62 $${P}_{Kipchoge,1}=14.988\cdot {v}_{i}^{2}+38.015\cdot {v}_{i}+91.509$$

63 $${P}_{Kiplimo,1}=13.720\cdot {v}_{i}^{2}+53.078\cdot {v}_{i}+48.861$$

This formation will be held for as long as the pacer with the lowest available energy (Koech) can keep up. The time spent, ${t}_{1}$, can be calculated by dividing his available energy by the instantaneous power output:

64 $${t}_{1}=\frac{3.295\cdot {10}^{6}}{{P}_{Koech,1}}$$

Evidently, this expression is a function of velocity $${v}_{i}$$. The distance covered during the first phase is simply $${d}_{1}={v}_{i}\cdot {t}_{1}$$ (also a function of $${v}_{i})$$. Before we can proceed to phase 2, we shall evaluate how much energy was consumed by Kiplimo and Kipchoge in the first phase, both of which are also functions of $${v}_{i}$$:

65 $${E}_{Kipchoge,1}={P}_{Kipchoge,1}\cdot {t}_{1}$$

66 $${E}_{Kiplimo,1}={P}_{Kiplimo,1}\cdot {t}_{1}$$

Phase 2: race continues at velocity ${v}_{i}$ and Formation 4, with Kiplimo as the only pacer and Kipchoge trailing behind. The total power outputs for the running positions have been calculated as functions of running velocity (Table 10):

67 $${P}_{Kipchoge,2}=14.841\cdot {v}_{i}^{2}+41.744\cdot {v}_{i}+77.723$$

68 $${P}_{Kiplimo,2}=14.611\cdot {v}_{i}^{2}+51.228\cdot {v}_{i}+46.766$$

The formation will be used for as long as Kiplimo can continue running at speed ${v}_{i}$. His limiting energy is the total available energy subtracted by the energy spent in phase 1:

69 $${E}_{Kiplimo,2}=3.592\cdot {10}^{6}-{E}_{Kiplimo,1}$$

The time spent, ${t}_{2}$, can be calculated by dividing his available energy by the instantaneous power output:

70 $${t}_{2}=\frac{3.592\cdot {10}^{6}-{E}_{Kiplimo,1}}{{P}_{Kiplimo,2}}$$

Once again, this expression is a function of velocity $${v}_{i}$$. The distance covered during the second phase is simply $${d}_{2}={v}_{i}\cdot {t}_{2}$$ (also a function of $${v}_{i})$$. The energy consumed by Kipchoge in phase 2 is:

71 $${E}_{Kipchoge,2}={P}_{Kipchoge,2}\cdot {t}_{2}$$

Phase 3: Kipchoge runs alone until the end of the race at a reduced speed ${v}_{f}$. His total power output is:

72 $${P}_{Kipchoge,3}=17.134\cdot {v}_{f}^{2}+24.301\cdot {v}_{f}+118.980$$

His total energy for that phase is the remaining energy after phases 1 and 2:

73 $${E}_{Kipchoge,3}=6.031\cdot {10}^{6}-{E}_{Kipchoge,1}-{E}_{Kipchoge,2}$$

The remaining distance to be covered is ${d}_{3}=42195-{d}_{1}-{d}_{2}$ m. If the final velocity is ${v}_{f}$, this distance will be covered in

74 $${t}_{3}=\frac{42195-{d}_{1}-{d}_{2}}{{v}_{f}}$$

The total race time is ${t}_{tot}={t}_{1}+{t}_{2}+{t}_{3}.$

This expression is a function of velocities ${v}_{i}$ and ${v}_{f}$. The total energy for phase 3 must be equal to the instantaneous power multiplied by the time:

75 $${E}_{Kipchoge,3}={P}_{Kipchoge,3}\cdot {t}_{3}$$

This is also an expression in terms of ${v}_{i}$ and ${v}_{f}$. In order to evaluate the velocity ${v}_{f}$ and the total time ${t}_{tot}$, the following steps are necessary:

- Define initial lower and upper limits for ${v}_{i}$, followed by the division of that interval into several smaller portions for greater accuracy;
- Substitute each possible value ${v}_{i}$ in the previous equation, resulting in an equation in ${v}_{f}$;
- Numerically solve for ${v}_{f}$. Substitute ${v}_{i}$ and ${v}_{f}$ in the appropriate expression and calculate ${t}_{tot}$;
- Plot ${v}_{f}$ and ${t}_{tot}$ against ${v}_{i}$. Determine the values for which the lowest total time is observed.

The results are presented in Fig. 21. The minimum time, 7197.028s (1:59:57), finally demonstrates the feasibility of the sub 2 h official marathon. In order to achieve this time, the protected phase would have to be run at 5.938 m/s and the unprotected phase at 5.754 m/s.

Additionally, the plot clearly shows the existence of a slight tolerance: if the unprotected phase is run at any speed between around 5.85 and 6.03 m/s, a sub 2 h result is still possible. However, the pace would have to be strictly controlled in order to achieve the optimal result.

### Application for the 2018 Berlin Marathon

The analysis was repeated for the 2018 Berlin marathon. Results are presented in Fig. 22. The minimum time, 7210.195s (2 h0 min10 s), represents an improvement of over one second on the previous best time. In order to achieve this time, the protected phase would have to be run at 5.918 m/s and the unprotected phase at 5.761 m/s.

Ultimately, in addition to leading to a sub 2 h pacing strategy for the 2022 Berlin marathon, the final strategy represents the possibility for an enormous improvement in Kipchoge’s performance for the 2018 Berlin marathon (for a 2:00:10 finish, 1 min and 39 s ahead of his 2:01:39 mark). The hypothesis of time improvement by running the protected phase at a higher speed is confirmed by these results.

## Abbreviation

CFD: Computational fluid dynamics
